# Glial-to-mesenchymal transition of tumor Schwann cells drives the genetic burden in MPNSTs from neurofibromatosis type 1 mouse model

**DOI:** 10.1126/sciadv.adt9210

**Published:** 2025-11-12

**Authors:** Katarzyna J. Radomska, Audrey Onfroy, Laure Lecerf, Bastien Job, Aurélien Beaude, Laura Sesma Sanz, Tatiana El Jalkh, Denis Thieffry, Patrick Charnay, Pierre Wolkenstein, Nicolas Ortonne, Fanny Coulpier, Piotr Topilko

**Affiliations:** ^1^ Mondor Institute for Biomedical Research (IMRB), INSERM, Créteil, France.; ^2^ Ecole Normale Supérieure, PSL Research University, CNRS, INSERM, Institut de Biologie de l’Ecole Normale Supérieure (IBENS), Paris, France.; ^3^ Gustave Roussy Institute of Oncology, Villejuif, France.; ^4^ National Phenotypic Screening Centre, School of Life Sciences, University of Dundee, Dundee, UK.; ^5^ Department of Dermatology, Reference Center for Neurofibromatosis, Henri-Mondor Hospital, AP-HP, Créteil, France.; ^6^ Department of Pathology, Henri-Mondor Hospital, AP-HP, Créteil, France.

## Abstract

There is currently no effective treatment for malignant peripheral nerve sheath tumors (MPNSTs), half of which result from malignant progression of neurofibromas (NFs) in patients with neurofibromatosis type 1 (NF1). NFs are due to biallelic loss-of-function of NF1, which negatively regulates the RAS pathway, in the Schwann cell lineage. We generated a conditional Nf1-mutant mouse model where NFs spontaneously transform into MPNSTs, faithfully recapitulating the human situation. Single-cell transcriptomic profiling demonstrated progression of NFs into MPNSTs, with a glial-to-mesenchymal transition. *Sox9* was identified as a marker of this transition and key player in tumor growth. The transition is followed by a loss of the tumor suppressor gene (TSG) *Cdkn2a* and acquisition of pathogenic variants of other TSGs. Finally, a proof-of-concept drug screen aimed at reducing *Sox9* expression in tumor cells identified 12 FDA-approved drugs. Notably, several of these agents target the RAS signaling cascade, suggesting that multi-targeted inhibition of this pathway may represent a promising therapeutic strategy against MPNSTs.

## Teaser

Tumor Schwann cells can undergo glial-to-mesenchymal transition and form malignant tumors in NF1 without genetic alterations.

## INTRODUCTION

Neurofibromatosis type 1 (NF1) is caused by loss-of-function (LOF) mutations in the *NF1* gene encoding a negative regulator of the RAS signaling pathway. Approximately half of NF1 patients develop plexiform neurofibromas (pNFs) along the nerve roots and bundles in soft tissues. pNFs are complex tumors composed of Schwann cells (SCs) mixed with other nerve fiber elements, including axons, fibroblasts (Fbs), blood vessels, mastocytes, and macrophages. Despite their cellular heterogeneity, pNFs arise from SC lineage that lose their remaining functional *NF1* [loss of heterozygosity (LOH)] copy, with permanent hyperactivation of RAS targets, including the mitogen-activated protein kinase (MAPK) pathway (*1*). pNFs are typically diagnosed in childhood and can transform into malignant peripheral nerve sheath tumors (MPNSTs), without effective therapy. MPNSTs account for 5 to 10% of soft tissue sarcomas, affecting adults between 20 and 60 years of age. About half of MPNSTs occur in NF1 patients, the remaining being radiotherapy-induced (10%) or sporadic (40%) (*1*). Malignant transformation of pNFs is preceded by a “premalignant” state, described as “dysplastic” or “atypical” NF (*2*), exhibiting abnormal SC morphology or increased cellular density. Recently, experts proposed a new terminology, classifying tumors into three categories: atypical NF (only atypical SCs), cellular NF (increased cellularity), and atypical neurofibromatous neoplasms of uncertain biologic potential (ANNUBP) (at least two atypical features like cellular atypia, hypercellularity, loss of pNF architecture, and rare mitoses) (*3*). Despite this terminology being now a reference, these tumors will herein be designated as dysplastic NFs (dyNFs). Although dyNFs are premalignant lesions of MPNSTs (*2*), definitive evidence of them being an early stage of pNF transformation into MPNST is lacking.

MPNST cells harbor complex genomic rearrangements, with NF1 invalidation necessary yet insufficient for MPNST development. MPNST development likely requires amplification of *PDGFRA*, *EGFR*, or *MET* oncogenes, LOF of *CDKN2A/B* or *TP53* tumor suppressor genes (TSGs), or genes encoding components of PRC2 epigenetic regulator, *SUZ12* and *EED1*. The genomic landscape of ANNUBP revealed a common loss of *CDKN2A/B*, without alterations in other genes mutated in MPNST (*4*). Despite seminal advances in molecular players and pathways controlling MPNST growth, the mechanisms governing stepwise progression of pNFs into dysplastic and malignant tumors remain poorly understood. This can be accounted for by interpatient molecular heterogeneity of MPNSTs and lack of corresponding animal models.

Over the past decade, numerous genetic tools have been developed to study NF1-associated MPNSTs, including genetically engineered mouse models with targeted inactivation of *Nf1* and various TSGs. To our knowledge, the model developed by Rhodes and colleagues (*5*), which uses Periostin-Cre (*Postn^Cre^*) to drive simultaneous inactivation of *Nf1* and *Arf* (an isoform of *Cdkn2a*), effectively recapitulates the progression of pNFs into MPNSTs or through an intermediate dysplastic stage (*5*). However, because *Postn* is expressed not only in SC lineage but also in nerve sheath Fbs, determining the precise cell(s) of origin and the tumor developmental dynamics remains challenging in this model. As previously observed, boundary cap (BC) cells, representing a neural crest–derived cell population transiently located at the dorsal root entry zone and ventral exit points of cranial and spinal nerves, give rise to SCs that migrate into the nerve roots and nerves innervating the skin (*6*). These are privileged sites where NFs develop in NF1 patients. Using a conditional mutation in *Nf1*, we specifically inactivated the gene in BC cells, showing that mutant mice develop cutaneous NFs and pNFs, which faithfully recapitulate molecular and cellular aspects of human pathology (*7*, *8*). Upon characterizing this animal model, we discovered that pNFs in mutant mice often transform into MPNSTs while this process undergoes a dysplastic stage, offering a robust system for characterizing dyNFs. Using combined genomics, single-cell transcriptomics, immunohistochemistry, and drug screening approaches, we found that malignant progression of pNFs is initiated by a fate transition of tumor SCs from a glial to a mesenchymal identity, followed by genetic alterations in TSGs. Drug screening studies using this model identified previously unidentified compounds targeting the RAS pathway, thereby underscoring its pivotal role.

## RESULTS

### Spontaneous progression of plexiform NFs to dysplastic and malignant tumors in *Prss56^Cre^, Nf1^fl/fl^* mutant mice

Our mouse model relies on the expression of *Prss56* gene, labeling a BC cell subpopulation that generates nerve root SCs and can elicit NFs (*5*). It consists of mice carrying the following allele combinations: *Prss56^Cre^*, *R26^tdTom^*, and *Nf1^fl/fl^* or *Prss56^Cre^*, *R26^tdTom^*, and *Nf1^fl/−^* (referred to as *Prss56^Cre^*, *Nf1^fl/fl^* or *Prss56^Cre^*, *Nf1^fl/−^* mice, respectively). The *R26^tdTom^* allele enabled identifying cells derived from progenitors having expressed *Prss56*, which permanently inactivated *Nf1*, yet activated tomato expression (TOM^+^). As reported, animals aged between 6 and 20 months develop various benign peripheral nerve sheath tumors (PNSTs), including cutaneous NFs (cNFs), and paraspinal and intraneural (subcutaneous) NFs, both defined as pNFs, with high penetrance (*7*). Regardless of their location, PNST types display a similar cellular composition with *Nf1*-null (TOM^+^) SCs, mixed with immune cells, nerve Fbs, and endothelial cells, within a dense extracellular matrix (ECM) (*7*).

**Fig. 1.**
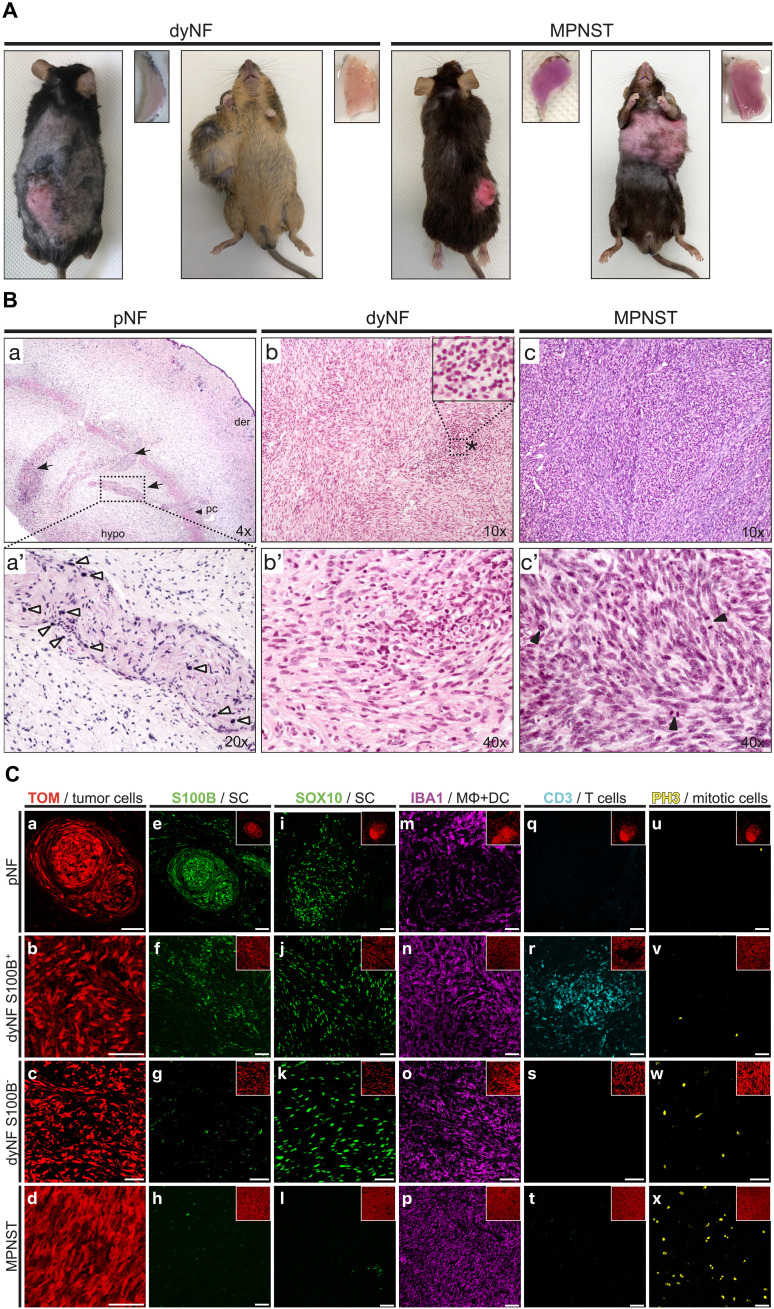
Spectrum of peripheral nerve sheath tumors in *Prss56*^Cre^, *Nf1*^fl/fl^ mice. (**A**) Gross anatomy of dyNF- and MPNST-bearing males. Dissected tumors are shown on the right. Note enhanced red shading of MPNSTs as compared to dyNFs, reflecting augmented densities of TOM^+^ neoplastic cells. (**B**) H&E staining of benign subcutaneous NFs (a, arrows), dyNFs (b), and MPNSTs (c) from *Prss56*^Cre^, *Nf1*^fl/fl^ mice (for H&E staining of MPNSTs in *Prss56*^Cre^, *Nf1*^fl/−^ mice, refer to fig. S1). High-magnification images are shown in the lower panel. Note increasing cellular densities and cellular/nuclear atypia in dyNF and MPNST versus pNF samples. Open arrowheads in (a′) indicate numerous mast cells infiltrating pNFs, but absent in dyNFs and MPNSTs. An asterisk in (b) indicates a cluster of mononuclear cells typically found in dyNFs (higher magnification is shown in the inset). Closed arrowheads in (c′) indicate frequent mitotic cells in MPNST. der, dermis; hypo, hypodermis; pc, panniculus carnosus muscle. (**C**) Sections through pNF, dyNF, and MPNST immunolabeled for TOM (TCs) (a to d), S100B (e to h) and SOX10 (i to l) (SC lineage), IBA1 (macrophages, DCs) (m to p), CD3 (T cells) (q to t), and PH3 (mitotic cells) (u to x). Insets show TOM staining of corresponding regions. Scale bar, 50 μm.

We followed mutant animals for longer periods, observing that some *Prss56^Cre^*, *Nf1^fl/fl^* and *Prss56^Cre^*, *Nf1^fl/−^* older mice also develop large, rapidly growing superficial TOM^+^ tumors in dorsum, limb, and cranial regions. Because of tumor size/ulceration or movement impairment, these animals require euthanasia at 13.1 ± 3.2 months (Fig. 1A and fig. S1A). Fast-growing tumors were only observed in group-housed mutant males with numerous bite marks resulting from their naturally aggressive behavior. Lack of tumors in males from the control *Prss56^Cre^*, *Nf1^+/+^* cohort, and in mutant males and females housed individually, suggested their development promoted by skin trauma or inflammation. Fast-growing tumors were never observed in nerve roots where paraspinal pNFs developed.

Gross histological evaluation of lesions (Fig. 1B and fig. S1B), using criteria established for human PNSTs (*3*), revealed them to display characteristics of either MPNSTs (pronounced hypercellularity and nuclear atypia, frequent mitotic figures (>4/mm^2^), apoptotic or necrotic cells, and cellular debris) or dyNFs, rather than of cellular NF and ANNUBP types [increased cellularity with fascicular growth pattern, loss of NF architecture, nuclear atypia, and rare mitotic figures (≤4/mm^2^)]. Numerous lesions exhibited a mixed architecture with varying proportions of both dyNF- and MPNST-like areas, reflecting an ongoing evolution toward malignancy (Fig. 1B, fig. S1B, and table S1). Among MPNST-like tumors, both low-grade (LG) [moderate hypercellularity and high mitotic index (>4 to ≤8)] and high-grade (HG) variants [marked hypercellularity; high mitotic index (>8 to ≤30)] were identified, with rare necrosis, in contrast to human MPNSTs (Fig. 1B and fig. S1B). The diagnostic delineation correlated with variations in immune infiltrates. Mast cells, easily identified by their round to oval morphology, granular cytoplasm, and characteristic dark purple staining, were profuse in benign lesions, but scarce/absent in dyNF- and MPNST-like tumors, with other mononuclear cells like macrophages and T lymphocytes being abundant in dyNFs versus both pNFs and MPNSTs (Fig. 1B, fig. S1B, and table S1). In dyNF-like tumors, T cells were aggregated around blood vessels (Fig. 1B and fig. S1B). Similar T cell–infiltrated dyNFs were observed in NF1 patients (*4*, *9*). Details on tumor phenotypes, gender, age at sacrifice, and genetic background of tumor-bearing mice are provided in table S1.

Immunohistochemical (IHC) analyses of pNFs and dyNF- and MPNST-like tumors (Fig. 1C) confirmed increased density and transformed (sarcomatous) morphology of Nf1-mutant (TOM^+^) cells, with augmented mitotic activity (PH3^+^), robust macrophages (IBA1), and scarce T cell (CD3^+^) infiltration in malignant versus benign/dysplastic lesions, as typical for human MPNSTs (*3*). Whereas most tumor SCs coexpressed the SC markers S100B and SOX10 in pNFs, these markers were not detected in MPNST-like tumors (Fig. 1C). Considering dyNF-like tumors, we identified two dyNF types, where tumor cells (TCs) were S100B^+^/SOX10^+^ (dyNF S100b^+^ type) or S100B^−^/SOX10^+^ (dyNF S100b^−^ type) (Fig. 1C). Having never observed TCs with a S100B^+^/SOX10^−^ signature, we hypothesized S100b silencing to precede that of Sox10, being possibly the earliest indication of malignant progression.

Moreover, dyNFs and MPNSTs similarly developed in both *Nf1* compound heterozygous (*Prss56^Cre^*, *Nf1^fl/−^*; 19 of 48 males = 39.6%) and floxed *Nf1* homozygous (*Prss56^Cre^*, *Nf1^fl/fl^*; 58 of 81 males = 71.6%) backgrounds. Tumor localization, growth rate, and histological characteristics were similar in both genotypes (Fig. 1, fig. S1, and table S1). Of note, 30% of *Prss56^Cre^*, *Nf1^fl/−^* males, within an age spectrum of 5 to 18 months, were found dead in cages or required euthanasia due to paralysis secondary to paraspinal NFs. Upon excluding premature deaths, the dyNF/MPNST incidence surged to 57.6%. The average age at sacrifice, dictated by tumor burden, was 12.6 ± 3.2 months and 14.1 ± 2.8 months for *Prss56^Cre^*, *Nf1^fl/fl^* and *Prss56^Cre^*, *Nf1^fl/−^* mutants, suggesting *Nf1* inactivation by Cre recombinase to be very efficient, with inactivation of one versus two alleles not making much of a difference.

All dyNFs and MPNSTs were located in the hypodermis, often infiltrating subcutaneous fat/muscle and extending into the dermis, raising the question of their precursor lesion’s nature. We identified two subcutaneous NF types, “nodular” and “diffuse,” in young mutant mice (fig. S1C). Nodular NFs corresponded to a well-demarcated accumulation of TOM^+^ SCs and tumor microenvironment, contributing to a local nerve enlargement (Fig. 1, B and C, and fig. S1C). Diffuse NFs showed nerve defasciculation with a diffuse growth pattern of TOM^+^ SCs (fig. S1C). As they could not be analyzed in detail, we were unable to assess whether the diffuse form corresponded to a more advanced-stage tumor development or distinct NF type. These two types of subcutaneous NF were reminiscent of those observed in human PNSTs (*10*). Conclusively, in our mouse model, subcutaneous NFs likely progress to dyNFs and MPNSTs, whose cellular characteristics faithfully recapitulate fast-growing PNSTs in NF1 patients.

### Single-cell dissection of cellular and molecular landscapes in benign, dysplastic, and malignant tumors

**Fig. 2.**
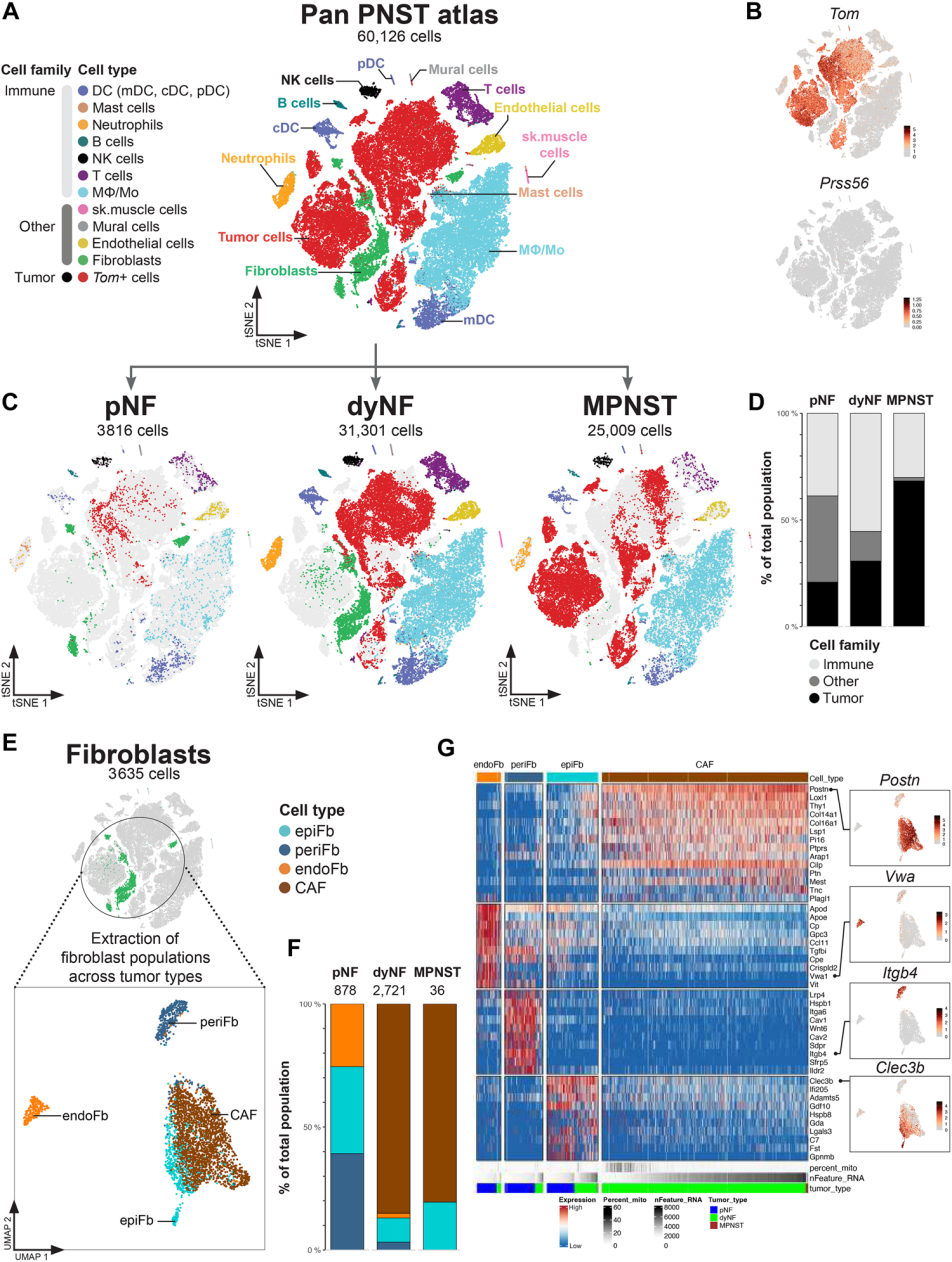
Single-cell profiling of *Prss56*^*Cre*^, *Nf1*^*fl/fl*^–derived tumors. (**A**) Pan-PNST transcriptomic atlas assembled from *Prss56*^*Cre*^, *Nf1*^*fl/−*^–derived pNF, dyNF, and MPNST datasets. Data are presented as tSNE projection to visualize cellular heterogeneity. Expression score–based annotation identified 12 cell populations. (**B**) Overlay of the pan-PNST atlas with expression of *Tom* and *Prss56*. Cells from the complete atlas are shown as light gray background. Cells are color-coded according to gene expression level. (**C**) Overlay of the pan-PNST atlas with transcriptomic data from each tumor type. Note the distinct locations of Fbs and TCs between different tumor types. (**D**) Dynamics of major cell populations throughout pNF-to-MPNST progression. (**E**) Heterogeneity of the Fb population, extracted from the pan-PNST atlas and visualized as UMAP plot. (**F**) Frequencies of Fb subtypes, according to tumor type. Cell numbers are shown on each bar’s top. (**G**) Heatmap representing differentially expressed genes for each Fb subtype. The expression of selected transcripts is shown on UMAP, on the right. CAF, cancer-associated Fbs; endoFb, endoneurial Fbs; epiFb, epineurial Fbs; periFb, perineurial Fbs.

To decipher how TC cellular composition, biological activities, and their microenvironment evolve during malignant transformation, we performed single-cell transcriptomic profiling of pNFs, dyNFs, and MPNSTs, issued from *Prss56^Cre^*,*Nf1^fl/fl^* mice, using the 10x Chromium platform (see Materials and Methods). We assembled all tumor samples (two pNF datasets, each containing a pool of 10 to 15 subcutaneous pNFs; nine dyNF and four MPNST datasets, each corresponding to individual tumors) into a comprehensive PNST (pan-PNST) transcriptomic atlas containing 60,126 cells. We used *t*-distributed stochastic neighbor embedding (tSNE) to visualize all cells in this unified dataset (Fig. 2A). We used a gene expression score–based cell type annotation method, enabling us to identify 12 distinct cell populations. Tom expression was a TC marker (Fig. 2, A and B, and fig. S2A). TCs (*Tom*^+^) were the dominant group. Since *Prss56* reactivation did not occur during pNF development and malignant progression (Fig. 2B), all Tom^+^ tumor SCs were considered originating from BC cells. The second group were immune cells (*Ptprc*^+^) comprising the myeloid lineage, including macrophages/monocytes (Mø/Mo) (*Adgre1*^+^/*Cd68*^+^), dendritic cells (DCs), neutrophils (*S100a9*^+^), and mast cells (*Tpsb2*^+^), and the lymphoid lineage, with T cells (*Cd3e*^+^), natural killer (NK) cells (*Ncr1*^+^), and B cells (*Cd79a*^+^). DCs exhibited heterogeneity, segregating into monocyte-derived DCs (mDCs) (*Cd209a*^+^/*Mgl2*^+^), classical DCs (cDCs) (Xcr1^+^), and plasmacytoid DCs (pDCs) (*Siglech*^+^). The remaining cells encompassed four distinct cell types: Fbs (*Lum*^+^/*Serping1*^+^), endothelial cells (*Pecam1*^+^), mural cells (*Pdgfrb*^+^/*Rgs5*^+^), with vascular smooth muscle cells and pericytes, and skeletal muscle cells (*Myl1*^+^).

We next split up the pan-PNST atlas by tumor type (Fig. 2, C and D) or dataset (fig. S2C) to explore how cellular composition and transcriptomic signatures evolve over the transformation steps. All cell populations were shared between all datasets (fig. S2C), suggesting that they are not tumor type specific, although their proportions varied among tumor types (Fig. 2, C and D). All cell types exhibited notable dynamics during transition. Unsurprisingly, the TC proportion progressively increased from the benign to the malignant stage, consistent with their relative proliferative activity (Fig. 1C and fig. S2B). In contrast, the proportion of immune cells peaked in the dysplastic phase versus both pNF and MPNST, while the proportion of all other cell types decreased with malignancy (Fig. 2D).

**Fig. 3.**
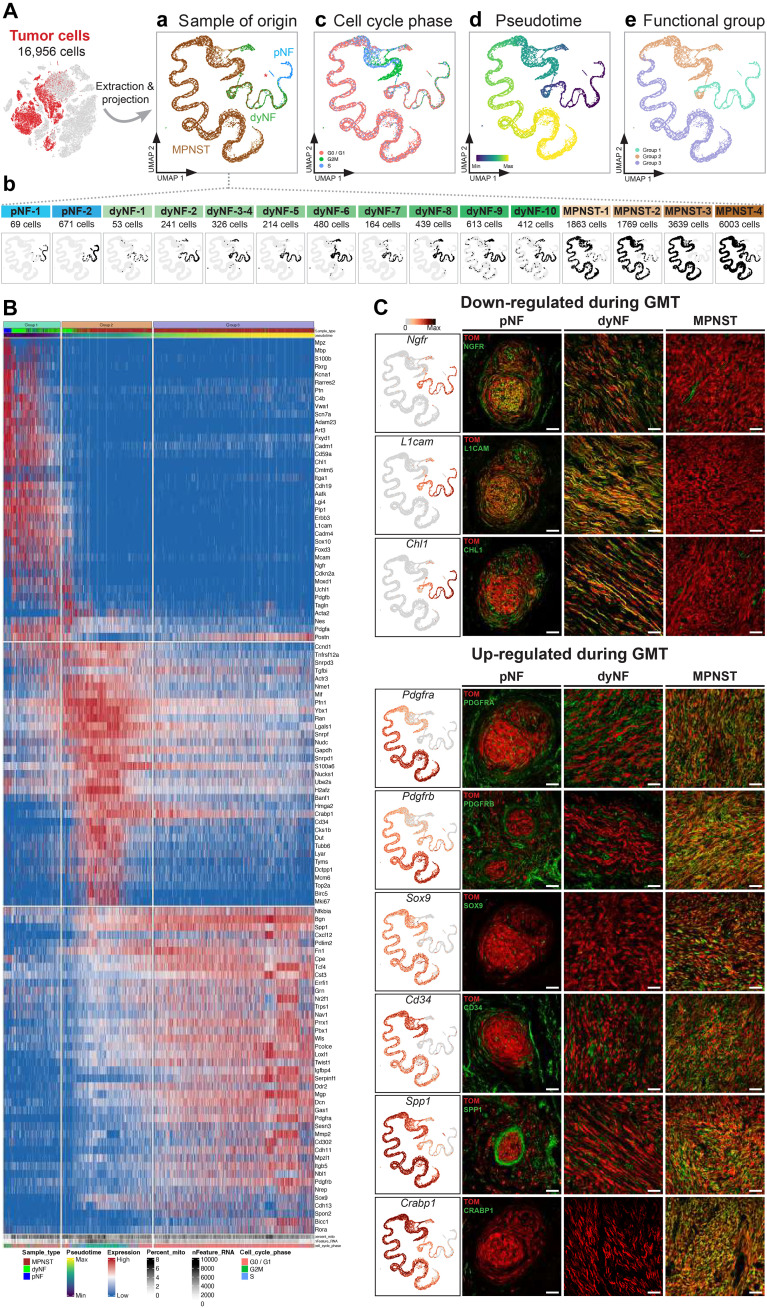
Transcriptional dynamics of TCs along malignant progression. (**A**) TCs were extracted and projected by combining DM and UMAP. On the resulting projection, cells were colored based on sample of origin (a), with individual contribution of pNF (*n* = 2), dyNF (*n* = 9), and MPNST (*n* = 4) datasets (b), cell cycle phase annotation (c), pseudotime inferred with slingshot (d), and functional group (e). In (a), the red star annotates myelinated SCs from pNF samples. See details in fig. S3A. In (b), datasets dyNF_3 and dyNF_4 were merged because they originated from the same primary tumor. (**B**) Heatmap showing transcriptomic changes in TCs during pNF-to-MPNST progression. Cells were ordered by increasing pseudotime values. The top annotation shows functional groups, tumor type, and pseudotime. Bottom annotation shows cell cycle phase (cell_cycle_phase) and quality metrics: proportion of expression level related to mitochondrial genes (percent_mito) and total number of genes detected by cell (nFeature_RNA). Genes were divided into three groups: glial signature, cell cycle signature, and mesenchymal signature. (**C**) Single-cell gene expression levels (left column) and protein detection of selected markers during GMT. Note that glial markers are coexpressed with TOM^+^ (tumor) cells in pNF and dyNF, but down-regulated in MPNST. Conversely, mesenchymal markers are expressed by non-TCs in pNF and dyNF and activated by TCs in MPNST.

The Fb population was divided into distinct, nonoverlapping subsets in the pNF versus dyNF and MPNST datasets (Fig. 3C). To analyze the Fb population’s diversity, the Uniform Manifold Approximation and Projection (UMAP) technique was used. UMAP delineated four distinct subsets (Fig. 3E), three of them corresponding to classical peripheral nerve sheath Fb subtypes, namely, epineurial Fbs (epiFb) (enriched for *Clec3b* and *Gpnbm2*), perineurial Fbs (periFb) (*Itga6*, *Itga4*), and endoneurial Fbs (endoFb) (*Apod*, *Cp*) (*11*). The fourth subset occurred only in dysplastic/malignant stages (Fig. 2, E and F), leading us to annotate them as cancer-associated Fbs (CAFs). Using differential gene expression analysis between classical Fb subtypes and CAFs, we identified CAF-enriched transcripts, including *Postn*, *Cilp*, *Loxl1*, *Thy1*, and *Pi16*, previously associated with CAF identity in various tumor types (Fig. 2G) (*12*, *13*). As CAF transcriptomic signature shared similarities among all three nerve Fb types, we could not determine from which specific type they originated. The absence of classical nerve-associated Fb in dyNF and MPNST can be interpreted as their robust reprogramming or dilution affecting MPNST cellular and transcriptomic landscapes. We compared single-cell transcriptomes of MPNSTs derived from *Prss56^Cre^*, *Nf1^fl/fl^* (*n* = 4) and *Prss56^Cre^*, *Nf1^fl/−^* (*n* = 3) mutant mice, creating a combined MPNST atlas comprising 47,653 cells (fig. S2, D and E). When analyzed by genotype, the cell types and transcriptomic profiles showed minimal variability. However, the immune (mainly macrophages/monocytes) fraction was underrepresented in tumors from *Prss56^Cre^*, *Nf1^fl/−^* mutants (fig. S2, F and G). Accordingly, the *Nf1* genetic background in the tumor microenvironment did not affect the cellular composition and transcriptomic activity of MPNSTs, underscoring the similarities between sporadic and NF1-associated MPNSTs and demonstrating that both models can effectively decipher the mechanisms governing their development.

### Transcriptional dynamics of *Nf1*-deficient TCs during benign-to-malignant progression

We sought to explore the transcriptomic heterogeneity within the TC population extracted from the pan-PNST atlas. Tom^+^ TCs were processed to remove low-quality cells. After batch-effect correction using FastMNN, 16,965 cells were projected by combining diffusion map (DM) and UMAP (Fig. 3A). DMs helped organizing cells by gradient of gene expression. Subsequent UMAP arranged almost all cells in a connected string, with dyNF cells among pNF and MPNST cells (Fig. 3Aa), pointing toward a progressive transformation recapitulating the global histological evolution. Only a small Tom^+^ cell subset originating from pNF datasets remained unconnected. These cells were myelinating SCs, as suggested by the expression of myelin-related transcripts like *Mbp*, *Mpz*, or *Pmp22* (fig. S3A). Individual sample contributions are shown in Fig. 3Ab. Note the gradual cell distribution shift, supporting a progressive transformation process. Consistent with our previous findings, most cycling cells originated from MPNST datasets (Fig. 3Ac).

To identify possible transition states, these cells were subjected to pseudo-temporal (pseudotime) ordering, using the Slingshot trajectory inference package on the batch-effect corrected space (*14*, *15*). Cells were organized along a linear pseudotime trajectory, passing through successive pNF, dyNF, and MPNST samples (Fig. 3A, a and d). We used the dynfeature package to identify genes whose expression varied according to pseudotime. We explored the expression patterns of top-ranked 2000 genes, visualizing on a heatmap 120 representative genes selected based on their high-level expression in specific (pNF, dyNF, or MPNST) tumor types, with cells ordered according to pseudotime (Fig. 3B). This analysis identified three main cell groups (Fig. 3Ae). To explore their underlying biology, we performed gene set enrichment analysis (GSEA) between each pair of groups (fig. S3B) (*16*, *17*).

Group 1 set was preferentially expressed in TCs from pNF and dyNF samples (early pseudotime) and enriched for roles in gliogenesis (fig. S3Ba). This set comprised numerous classical SC-related markers like *Plp1*, *Sox10*, *S100b*, *Mcam*, *L1cam*, *Ngfr*, *Cdh19*, and *Foxd3*. Group 2 set (mid-pseudotime) was enriched in functional categories related to cell cycle regulation, exemplified by numerous proliferation-related transcripts like *Cenpa*, *Mki67*, *Top2a*, or *Birc5*. Group 2 proliferative cells exhibited high ribosomal RNA content, mainly derived from MPNST samples, yet additionally including a small dyNF cell subset (Fig. 3A, a and c, and fig. S3Bb).

Group 3 set was up-regulated in the outermost trajectory (late pseudotime), featuring part of the MPNST cells and being enriched with mesenchymal-like signature. Enrichment was observed in categories linked to ECM degradation/reorganization, cellular migration, hypoxia, and apoptosis (fig. S3Bc), ECM and adhesion molecules (*Dcn*, *Mgp*, *Bgn*, *Vcan*, *Cd34*, *Col3a1/2*, *Fn1*, *Mmp2*, *Cdh11/13*, and *Vcam1*), growth factors and receptors (*Pdgfra/b*, *Fgfr1*, and *Igfbp3/4/6*), stress and metabolism mediators (*Gas1*, *Nupr1*, and *Errfi1*), and transcription factors, including *Prrx1*, *Twist1*, *Sox9*, and *Snai1*, as known drivers of epithelial-to-mesenchymal transition (EMT) (Fig. 3B) (*18*). EMT was previously implicated in malignant progression in epithelial cancers by promoting acquisition of invasive cell phenotype, metastasis, and resistance to chemo- and immunotherapy (*19*). According to our findings, a similar mechanism, which we define as glial-to-mesenchymal transition (GMT), may drive malignant progression of dyNFs, supported by the morphological transition of TOM^+^ cells from spindle-shaped, bipolar SCs in pNFs to large-size, multipolar, Fb-like TCs in MPNSTs (Fig. 1C, a to d). Validation of the down- or up-regulation of selected markers during pNF-to-MPNST progression is presented in Fig. 3C.

We analyzed in detail the transcriptomic signature of TCs from dyNFs to identify characteristic markers (Fig. 3B). In all dyNF cells, we observed the extinction of myelinating SC-specific gene expression, along with decreasing expression of mature SC-associated markers (*S100b*, *Kcna1*, and *Cdh19*). In contrast, SC lineage markers (*Sox10*, *Erbb3*, and *Plp1*) were still expressed. Unexpectedly, we identified a group of genes whose expression was restricted to dyNFs (*Moxd1*, *Uchl1*, *Tagln*, and *Acta2*). Their biological impact remains unknown. No mesenchymal markers (*Crabp1*, *Sox9*, and *Spp1*) were detected in TCs from dyNFs. Using two markers, *Sox10* and *Sox9*, was thus sufficient to distinguish between dysplastic (*Sox10*^+^/*Sox9*^−^) and malignant (*Sox10*^−^/*Sox9*^+^) TCs. The additional use of *S100b* rendered it likely possible to discriminate between early-stage (*S100b*^+^) and late-stage (*S100b*^−^) dyNFs (Fig. 1C). Recently, the biallelic loss of *Cdkn2a*, a negative cell cycle regulator, was considered an early event driving MPNST development in NF1 patients (*4*). Consequently, while *Cdkn2a* was expressed in TCs from pNFs and almost all dyNFs, complete expression loss occurred in MPNSTs (Fig. 3B).

Accordingly, the notion of cellular malignant progression from pNF to MPNST appears robust, being characterized by gradual glial marker losses, along with robust activation of mesenchymal markers and increased proliferation. The transcription factors *Sox10* and *Sox9* proved sufficient to classify TCs as dysplastic or malignant.

### *Nf1-KO* mice and NF1 patients display similar transcriptome landscapes during malignant progression

We investigated if transcriptional signatures observed upon pNF-to-MPNST progression reflected those of NF1 patients. Therefore, we generated a single-cell transcriptomic atlas from patient-derived dyNFs (*N* = 3) and MPNST (*N* = 1) (table S2), integrating it into publicly available datasets of dyNF (*N* = 1) (accession no. GSE165826), pNFs (*N* = 9), and MPNSTs (*N* = 4) (accession no. GSE179033), creating a unified tSNE representation (pan-PNST atlas) comprising 78,518 cells (fig. S4A). Notable heterogeneity in cell composition and transcriptomic signatures across patient samples was observed, possibly accounted for by several factors: (i) varying intervals between surgical tumor removal and RNA extraction, (ii) frequent incomplete tissue dissociation, and (iii) interpatient variations (tumor size, location, and developmental stage, including HG-MPNSTs). Cells were annotated for cell types using the score-based cell annotation established in our mouse PNST atlas. This strategy effectively separated TCs from other cell types. Since both MPNST TCs and Fbs display high-level expression of mesenchymal markers, we further evaluated their differences using the Infercnv package. TCs exhibited more inferred large-scale chromosomal copy number variations (CNVs) versus Fbs, validating our gene-based annotation.

Once cell types were successfully annotated, the global cellular compositions of pNFs, dyNFs, and MPNSTs in patients resembled those of our mouse model, including an increased TC proportion, decreased Fb number, and macrophage abundance (fig. S4A). We then extracted TCs to generate a UMAP (fig. S4B). A mouse-like transition in *SOX10*/*SOX9* expression was observed across pNF, dyNF, and MPNST samples (fig. S4, C and D). In TCs from pNFs and dyNFs, *SOX10* was coexpressed with other glial markers like *S100B*, *SCN7A*, *CDH19*, *CHL1*, and *L1CAM*. Conversely, *SOX9* was activated in MPNSTs along with mesenchymal markers such as *CD34*, *PDGFRA*, and *PDGFRB*.

Overall, cell type annotations across both species underscore the robustness of our mouse model, which accurately reproduces the transcriptomic profiles of human PNSTs. An interesting difference emerged: Differential gene expression analysis between patient TC and Fb populations identified delta-like noncanonical Notch ligand 1 (*DLK1*) as the most selective marker for MPNST TCs. *DLK1* was not expressed in MPNST-associated Fbs (fig. S4A) or in TCs from pNFs and dyNFs. In contrast, *DLK1* expression was not detected in our mouse pan-PNST atlas. As *DLK1* encodes a transmembrane protein, we propose using it to differentiate and separate TCs from Fbs in human MPNSTs.

### dyNF allografts progress into MPNSTs

To demonstrate that MPNSTs originated from dyNFs through malignant progression, we transplanted TCs isolated from two dyNFs, diagnosed by their histological and transcriptomic signatures, into nude recipient mice (fig. S5). In both primary tumors, nearly all Tom^+^ TCs expressed *Sox10* and *Cdkn2a*, but not *Sox9*. Grafted cells generated fast-growing tumors collected 8 weeks later. Their transcriptomes, analyzed using single cell RNA sequencing (scRNA-Seq), were compared with those of primary tumors (fig. S5A, a to c). In each grafted tumor, we observed a mixture of *Sox10*^+^/*Sox9*^−^ and *Sox10*^−^/*Sox9*^+^ TCs, suggesting GMT within the tumor population (fig. S5B). Using the same methods as for TC profiling from the pan-PNST atlas, both primary and transplanted TCs were ordered by pseudotime values (fig. S5Ad), with gene expression profiles on a heatmap (fig. S5C). The transcriptomic signature of transplanted TOM^+^ TCs resembled that of primary MPNSTs (Fig. 3B and fig. S5C). Whereas *Cdkn2a* was maintained in *Sox10*^+^ cells, most *Sox9*^+^ TCs were *Cdkn2a*^−^, and all *Cdkn2a*^−^ cells were *Sox9*^+^, raising the possibility that *Sox9* activation occurs before *Cdkn2a* loss. These data support the preserved malignant transformation process in our xenograft model (fig. S5D), underlining the hypotheses of a GMT and dyNF origin of MPNST cells.

### Progression of NFs into MPNSTs is associated with biallelic *Cdkn2a* loss in TCs

To investigate whether malignant NF progression correlates with acquiring specific genomic alterations, we performed whole-exome sequencing (WES), and copy number variant (CNV) and single-nucleotide variant (SNV) analyses, on tumor biopsies from *Prss56^Cre^*, *Nf1^fl/fl^* (*n* = 7) and *Prss56^Cre^*, *Nf1^fl/−^* (*n* = 6) mutant mice. Spleen samples from the same individuals served as internal controls. All 13 tumors were classified as MPNSTs, using histological evaluation by experienced pathologists and TC scRNA-Seq signature, with detailed characteristics provided in table S1.

**Fig. 4.**
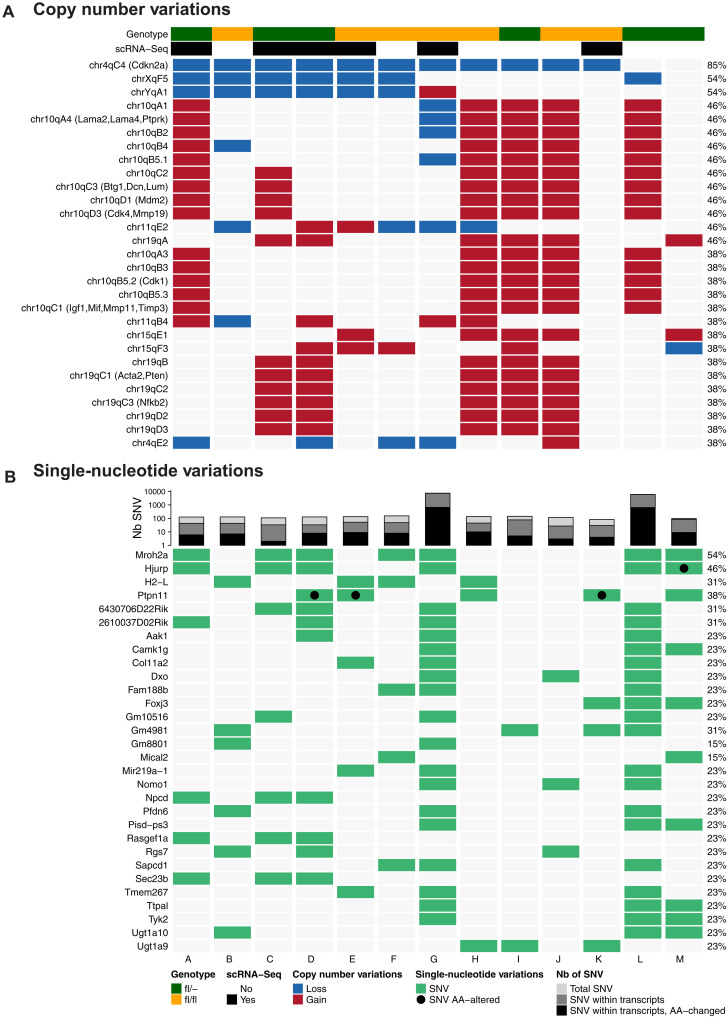
Genomic analysis of TCs isolated from mutant mouse MPNSTs. (**A**) Oncoplot showing CNV of TCs. Each column corresponds to an independent primary tumor. Top annotation shows *Nf1* genotype of mice and if the tumor has been characterized by scRNA-Seq. The rows below correspond to chromosomes with either gain or loss of genetic material. Note that 11 of 13 tumors show loss of parts of chromosome 4 carrying the *Cdkn2a* allele. (**B**) Oncoplot showing SNV of TCs. Columns are matched with (A). The top line represents the SNV number for each tumor. A selected list of genes carrying SNVs in different samples is shown in the rows below. SNVs with described pathogenic variants are indicated by a black dot. Note the presence of a pathogenic variant of *Ptpn11* in 3 of 13 tumors. Frequencies of genetic events (CNV and SNV) are shown on the right. The color code for legends is shown below.

On the basis of CNV analysis, 11 of 13 tumors presented deletions of portions of chromosome 4 covering *Cdkn2a* (Fig. 4A) (*3*). For 10 of these 11 tumors, deletion extended to neighboring regions containing *Cdkn2b*, *Mtap*, and *Ifn*(s) loci, whose co-deletion with *Cdkn2a* was reported in patient MPNSTs (*20*). Other rare genomic rearrangements comprised amplifications of portions of chromosomes 10, 15, and 19, including *Ptprk*, *Cdk1/4*, *Pten*, and *Acta2*, also described in MPNSTs from NF1 patients (*21*, *22*). SNV across all tumors exhibited a low number of variants (Fig. 4B). Neither CNV nor SNV analyses detected mutations in known TSGs like *Suz12*, *Eed*, and *p53*, commonly linked with sporadic and NF1-associated HG-MPNSTs (*4*). CNV and SNV analyses of the remaining two MPNSTs (both *Prss56^Cre^*, *Nf1^fl/−^*) revealed no genetic alterations in *Cdkn2a* or any other MPNST-associated TSG, with a low number of variants. Strikingly, 3 of 13 tumors exhibited pathogenic variants in *Ptpn11* (encoding SHP-2) found in 50% of Noonan syndrome patients (Fig. 4B) (*23*). Recent studies revealed *PTPN11* loss combined with *NF1* inactivation to promote malignancy of *NF1*-deficient cells (*24*).

As shown, biallelic loss of *Cdkn2a* is the most common and probably the earliest genomic event occurring in MPNSTs. We did not identify *Cdkn2a* loss in 2 of the 13 analyzed tumors, thus being probably not mandatory for tumor progression.

### Successive MPNST grafting leads to pathogenic variant acquisition in TCs

**Fig. 5.**
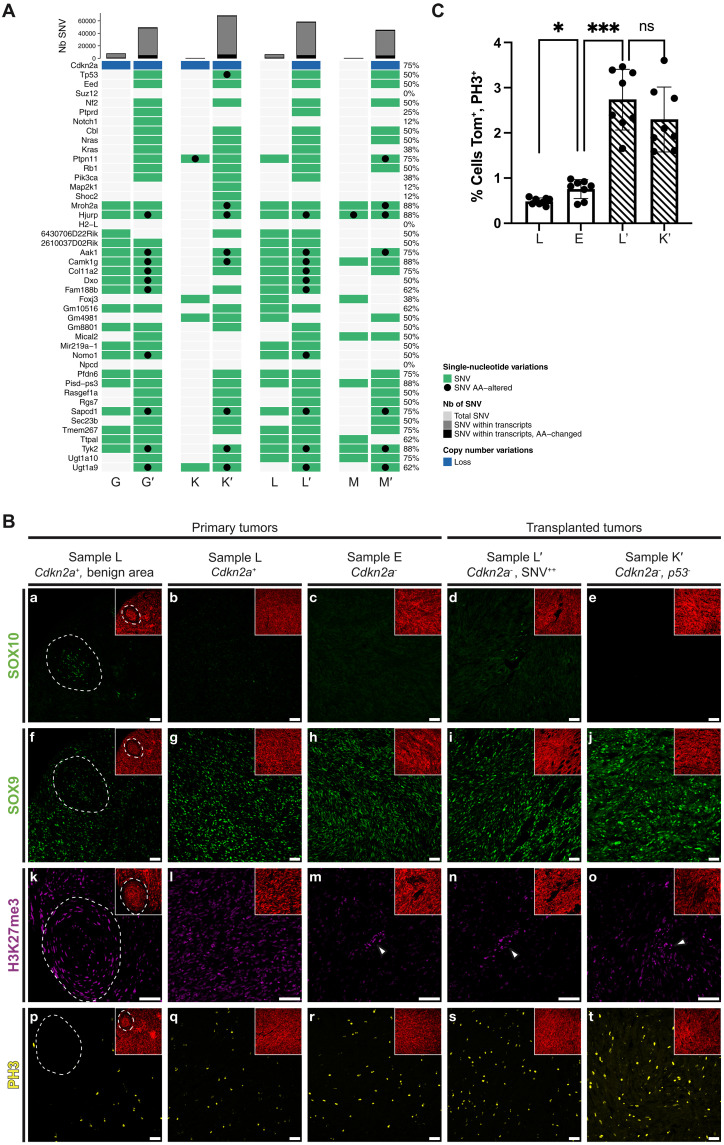
Genomic and IHC characterization of TCs from sequentially transplanted MPNSTs. (**A**) Oncoplot showing WES results of TCs. Each pair of columns corresponds to the primary (left) and its associated transplanted (right) tumor. The top line represents the SNV number for each tumor. The row below corresponds to CNV result at *Cdkn2a* locus. The remaining rows correspond to a selected list of genes carrying SNVs, with pathogenic variants described indicated by a black dot. Frequencies of genetic events and color code for legends are shown on the right. (**B**) Sections through primary and transplanted MPNSTs, immunolabeled for SOX10 (a to e), SOX9 (f to j), H3K27me3 (k to o), and PH3 (p to t). Insets in (a) to (t) show TOM labeling of the corresponding lesions. Scale bar, 50 μm. Each column depicts sections from the same tumor sample. Dashed circle delineates benign areas (nerves) as internal control. Note increased H3K27me3 staining (m to o) in TOM^−^ area pointed by arrowheads (m to o). (**C**) Quantification of TOM^+^, PH3^+^ cycling TCs in each tumor. Nonparametric *t* test *P* value: ns: nonsignificant, *P < 0.05, **P < 0.01, ***P < 0.001.

We sought to determine whether the low number of variants and the absence of pathogenic variants in TSGs like *Suz12*, *Eed*, and *Trp53*, considered to be a genetic hallmark of MPNSTs, were either due to our model’s particularity or rapid tumor growth, dictating early animal sacrifice within 2 to 3 weeks. Therefore, we performed four successive transplantations of TCs isolated from two *Cdkn2a*^−^ and two *Cdkn2a*^+^ MPNSTs. Approximately 100,000 TOM^+^ TCs from each primary tumor were grafted subcutaneously in nude recipients. After 2- to 4-week monitoring, tumors were extracted and dissociated, with a fraction (10,000 cells) regrafted using the same procedure. After four passages, tumors were processed for WES followed by CNV and SNV analyses, with results detailed in Fig. 5.

First, we observed accelerated growth of all tumors across successive passages, suggesting the acquisition of additional genetic aberrations promoting TC proliferation. Genomic analyses confirmed a notable increase in variants in all transplanted tumors (Fig. 5A). Specifically, in both *Cdkn2a*^+^ transplanted MPNSTs, a loss of this marker was identified. In tumors from mice grafted with *Cdkn2a*^−^ TCs, variants were found in *Trp53*, *Eed*, *Pdgfra*, and *Pdgfrb*, including a pathogenic variant of *Trp53* (c.515G>A, p.Arg172His) (*25*).

To assess the impact of these genetic aberrations, comparative IHC profiling of primary and transplanted tumors was performed (Fig. 5B). All samples showed a loss of glial markers SOX10 (Fig. 5B) and S100B, with the acquisition of GMT marker SOX9 in TCs (Fig. 5B). The rare SOX10^+^ labeling corresponded to TOM^−^ SCs from tumor-infiltrating nerves (Fig. 5Bd). Phospho–histone H3 (PH3) labeling revealed *Cdkn2a* inactivation in TCs, with a high proportion of variants including the pathogenic *Trp53* variant, correlating with increased proliferative activity (Fig. 5, B and C).

We performed immunolabeling for the tri-methylation of lysine 27 of histone H3 (H3K27me3), an epigenetic mark indicating polycomb repressive complex 2 (PRC2) activity. PRC2 catalyzed this epigenetic mark, commonly associated with gene repression (*26*). H3K27me3 proved often lost in MPNSTs from NF1 patients (*20*). H3K27me3 labeling was substantially reduced in TOM^+^ TCs from *Cdkn2a*^−^ primary and transplanted tumors versus sparse TOM^−^ SCs from the same samples (arrowheads in Fig. 5B, m to o) and *Cdkn2a*^+^ primary tumors (Fig. 5Bl).

In summary, successive grafting of MPNST cells resulted in rapidly growing tumors with progressive accumulation of genetic aberrations, including pathogenic variants in *Trp53*. In most tumors, the initial event involved a biallelic *Cdkn2a* loss, followed by an increased number of variants and emerging pathogenic variants in other TSGs. This increased genetic burden correlated with accelerated proliferation and reduced PRC2 activity in TCs.

### *Sox9* knockdown impedes MPNST growth in vivo

**Fig. 6.**
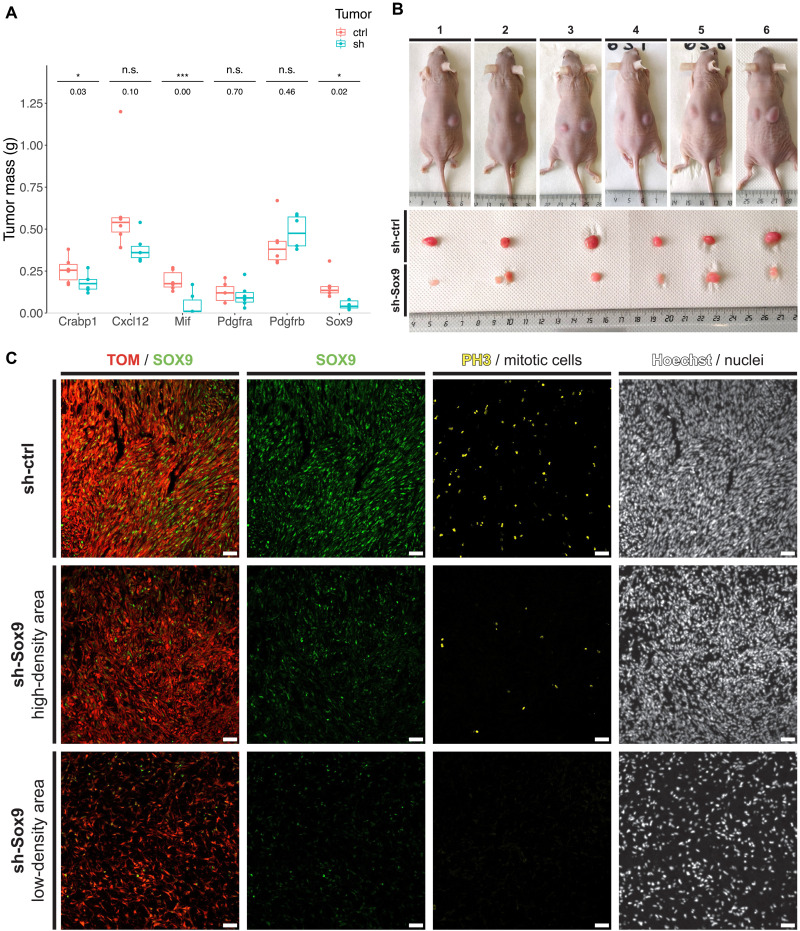
*Sox9* KD impedes MPNST growth in vivo. (**A**) Graph representing tumor mass comparison between tumors transfected with control shRNA (ctrl, red) and shRNA directed against the indicated candidate genes (sh, blue). Each dot corresponds to one transplanted tumor. Nonparametric *t* test *P* value: ns: nonsignificant, *P < 0.05, **P < 0.01, ***P < 0.001. (**B**) Macroscopic view of nude mice 2 weeks after subcutaneous injection of primary TCs, transfected with control shRNA (right flank) or anti-Sox9 shRNA (left flank), along with dissected tumors on bottom part. (**C**) IHC of tumors developed in nude mice transplanted as indicated on the left (sh-ctrl or sh-Sox9, two areas) and labeled for TOM (TCs), SOX9, PH3 (mitotic cells), and Hoechst (nuclei). Scale bar, 50 μm.

To investigate the role of genes whose activation in TCs correlates with GMT, we selected six candidates (*Sox9*, *Pdgfra*, *Pdgfrb*, *Mif*, *Cxcl12*, and *Crabp1*) based on their strong and sustained activation during GMT in both mouse and patient tumors (Fig. 3B and fig. S4C). Some of them, including *Sox9*, are known to be involved in the development of MPNSTs and other cancers (*25*, *27*, *28*). Each candidate gene was inactivated in primary mouse MPNST cells with a *Sox10*^−^/*Sox9*^+^/*Cdkn2a*^−^ signature using short hairpin RNA (shRNA)–mediated gene knockdown (KD) (see Materials and Methods). The efficacy of stable KD, measured by quantitative real-time polymerase chain reaction (qRT-PCR), varied among the target genes (table S3). For each candidate, at least two clones were used for transplantation into athymic nude mice. Approximately 10,000 TCs transduced with target shRNA or an empty vector (control; sh-ctrl) were injected into the left and right flanks, respectively, of six nude mice, and tumor growth was monitored over 2 weeks. All grafted mice were then sacrificed, and both the shRNA-silenced and control tumors were dissected, weighed, and measured. Despite heterogeneity in tumor mass within each group, substantial growth differences occurred when *Crabp1*, *Mif*, and *Sox9* were targeted, highlighting their role in tumor growth (Fig. 6A).

*Sox9* is particularly interesting due to its pivotal role in regulating genes governing EMT. A 60% reduction in *Sox9* mRNA levels before grafting was shown to significantly impede tumor growth (Fig. 6, A and B). We observed that while sh-control tumors appeared relatively homogeneous, characterized by densely packed Tom^+^ TCs, sh-Sox9 tumors exhibited marked heterogeneity, with regions of both high and low TC density (Fig. 6C). IHC analysis of two sh-control and two sh-Sox9 tumors using markers for TOM (TCs), SOX9, and PH3 (mitotic index) confirmed a substantial reduction in SOX9 protein levels and mitotic activity in sh-Sox9 tumors compared to controls (Fig. 6C). TC density was notably decreased, consistent with the observed reduction in overall tumor volume. While the expression of certain mesenchymal markers such as SPP1 and PDGFRB remained unchanged, the expression of CRABP1, MIF, PDGFRA, and CD34 was substantially reduced, suggesting diminished malignant potential (fig. S6). We did not report the reactivation of the glial markers S100B and SOX10. sh-Sox9 tumors also exhibited a marked decrease in expression of the vascular marker PECAM, indicating reduced density of blood vessels and possible hypoxia (fig. S6). Overall, these findings reinforce *Sox9*’s relevance in MPNST progression and its potential as a therapeutic target for preventing GMT.

### Drugs targeting the RAS pathway efficiently inhibit *Sox9* expression in TCs from mouse MPNSTs

**Fig. 7.**
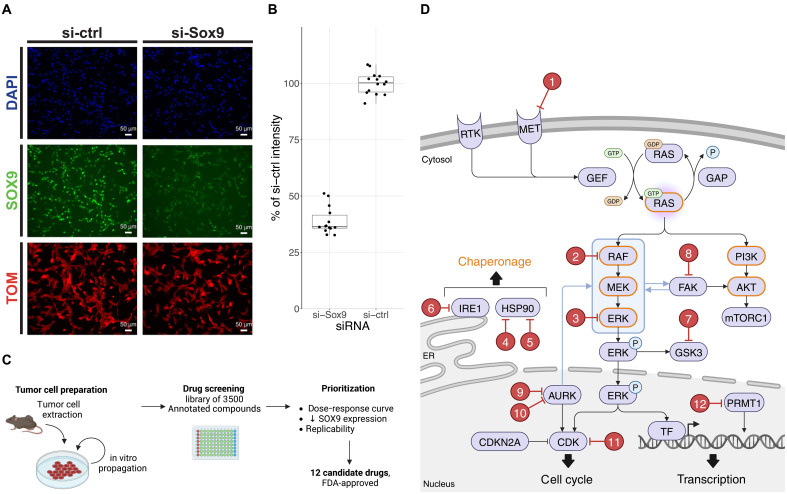
Sox9 expression in TCs could be driven by the Ras signaling pathway. (**A**) TC culture from primary MPNST, transfected with control siRNA (si-ctrl) or siRNA against *Sox9* transcript (si-Sox9), immunolabeled for DAPI (nuclei), SOX9, and TOM (TCs). Note decreased SOX9 labeling upon exposure to anti-Sox9 siRNA. Scale bar, 50 μm. (**B**) Quantification of SOX9 staining intensity in TOM^+^ cells, transfected with control siRNA (si-ctrl) or siRNA against *Sox9* transcript (si-Sox9). (**C**) Experimental design of in vitro screening of 3500 annotated compounds, leading to the identification of 12 FDA-approved candidate drugs. (**D**) Representation of molecular targets for 12 candidate drugs in relation with Ras signaling pathway. IRE1 and HSP90 are involved in the protein chaperonage with orange bold border. Correspondence between drug number and identity is attached in table S4. RTK, receptor tyrosine kinase; TF, transcription factor; GEF, guanine nucleotide exchange factor; GAP, guanosine triphosphatase–activating protein. Created in BioRender. Coulpier, F. (2025) https://BioRender.com/r79ogro.

As *Sox9* inhibition in mouse MPNST cells reduced tumor growth after transplantation in nude mice, we hypothesized that compounds inhibiting Sox9 expression would impede MPNST development. To identify such molecules, we conducted high-content screening of a biologically active compound library using a modified version of our primary MPNST culture system (see Materials and Methods). The cultured TCs (*Sox10*^−^/*Sox9*^+^/*Cdkn2a*^−^) were derived from primary mouse MPNSTs. In initial experiments, amplified TCs were transfected with small interfering RNA (siRNA) targeting *Sox9* to measure inhibition efficacy, showing a ≥50% reduction in SOX9 protein levels (Fig. 7, A and B). Drug screening was conducted with the European Drug Discovery Consortium (EuLeadFactory), using a library of 3500 biologically active annotated compounds, following the schematic design presented in Fig. 7C. Overall, 228 compounds leading to a >20% reduction in nuclear SOX9 signal intensity versus a dimethyl sulfoxide (DMSO) control were identified. These molecules were tested in dose-response curves, ranging from 30 nM to 30 μM. Using a nuclear SOX9 signal intensity reduction cutoff <50% of the DMSO control, we identified 12 candidates, all being U.S. Food and Drug Administration (FDA)–approved drugs (table S4). Measuring their impact on TC proliferation was challenging due to the short (24-hour) incubation time and induced morphology changes, rendering automatic cell counting inaccurate. Nevertheless, several of 12 compounds are known inhibitors of distinct elements of the RAS signaling pathway, including the MAPK pathway, for which NF1 is a negative regulator (Fig. 7D). Despite the presence, in the library, of numerous inhibitors of other signaling pathways (Wnt, HH, TGF, Notch) involved in the terminal phase of malignant transformation, none of them was identified as potent inhibitor of *Sox9* expression, reinforcing our findings’ relevance.

These results clearly validate our culture system for identifying drugs targeting MPNSTs. As a proof of concept, we used the GMT marker *Sox9*, since its inhibition delays tumor growth in vivo. *Sox9* expression in TCs is likely regulated by the RAS signaling pathway, thus being possibly a promising therapeutic target for combating MPNSTs.

## DISCUSSION

### A *Nf1*-KO mouse model faithfully recapitulating sporadic and NF1-derived MPNST pathogenesis

We herein characterized an NF1 mouse model that faithfully recapitulates the stepwise progression of MPNSTs. In this system, tumors arise on both wild-type and *Nf1^+/−^* genetic backgrounds, demonstrating that malignant transformation requires the acquisition of additional mutations in either setting. These results indicate that future therapeutic agents are expected to show comparable efficacy across both tumor contexts.

In NF1 patients, nodular pNFs are at risk of malignant progression through a dyNF intermediate step (*29*). However, this pathway has not been firmly demonstrated, nor has the stepwise progression of dyNFs into MPNSTs deciphered. Moreover, nodular and diffuse pNFs were not identified and characterized in non-NF1 patients. In our model, both nodular and diffuse subcutaneous pNFs occurred. Recently, scRNA-Seq analyses performed on nodular and diffuse pNFs revealed the presence of CAFs (Fb population transiently present in dyNFs) and of tumor SC expressing dyNF markers (*Moxd1* and *Uchl1*) only in nodular NFs (unpublished results). Thus, nodular NFs are likely at the origin of dyNFs. As nodular NFs in NF1 patients are small and often asymptomatic, they could remain undetectable in non-NF1 individuals and, in some situations, become malignant. By grafting dyNF-derived TCs in nude mice, we demonstrated that they are at the origin of MPNSTs. In our mouse model, some nodular subcutaneous pNFs progressed into dyNFs, which transformed into MPNSTs, completely illustrating human MPNST pathogenesis.

Despite the numerous previous NF1 mouse models leading to pNF development, their frequent spontaneous progression into MPNSTs, as in our model, is unprecedented. This highlights that BC cells are likely at the origin of MPNSTs. Recent studies identified rare glial stem-like cells (GSCs) in peripheral nerves, characterized by spherogenic capacity and Nestin expression, as potential progenitors of MPNSTs (*30*). Consistently, we revealed BC-derived glia from adult subcutaneous nerves to comprise spherogenic GSCs (*6*), supporting that these cells might give rise to MPNSTs. Further characterizing these cells will be decisive in determining the cancer stem cell origin of MPNSTs.

### GMT as a step to malignancy and potential therapeutic target

Although substantial progress in the cellular and molecular MPNST characterization was made over the last decade, the mechanisms underlying malignant initiation and progression remain poorly understood. This is largely due to their asymptomatic nature, being undetectable at their early malignancy phase, and the lack of animal models faithfully recapitulating this process. In our model, NFs frequently progressed to dyNFs, which evolved into MPNSTs, with transcriptional signatures sharing commonalities with MPNSTs from NF1 patients. This genetic tool appears thus appropriate to decipher the process of malignant transformation in both species. Using an scRNA-Seq approach, we built a comprehensive PNST transcriptomic atlas of more than 60,000 cells, including TCs and their microenvironment, such as pNFs, dyNFs, and MPNSTs. This is a major asset for the scientific community interested in MPNST pathogenesis and sarcoma development. Its analysis already revealed pNF-to-dyNF evolution to involve progressive extinction of SC markers, yet with preserved expression of SC lineage marker *Sox10*. Contrarily, the dyNF-to-MPNST transition was characterized by *Sox10* expression loss and, in parallel, robust activation of mesenchymal markers. These include *Sox9* and several other transcription factors being involved in EMT and regulated by *Sox9* upon development and cancer formation (*31*). In analyzed tumors, TCs never coexpressed *Sox10* and *Sox9*, suggesting a likely epigenetic mechanism responsible for simultaneous extinction of *Sox10* and activation of *Sox9* and other mesenchymal markers. We observed an increased TC proliferative activity, with morphological transition from thin bipolar glial cells to large, flat multipolar mesenchymal cells, without genetic alterations. This argues in favor of an abrupt GMT, being possibly pivotal in malignant progression. Combined ATAC-Seq/RNA-Seq of TCs during the GMT is particularly useful to investigate the underlying mechanisms. In all TCs analyzed, *Cdkn2a* loss only occurred when *Sox9* was activated, suggesting this latter event to precede genetic alterations. In dyNFs, we found GMT to occur without genetic alterations in *Cdkn2a* and other TSGs. This is not due to any of our model’s particularities, as *Cdkn2a* was rapidly altered/lost in TCs following primary tumor transplantation in nude mice. Accordingly, genetic alterations are not mandatory for GMT, whereas GMT might promote genetic instability, starting with *Cdkn2a* loss followed by various additional alterations. Blocking GMT might thus prevent malignant transformation and reverse TCs to their initial glial fate.

### Multi-targeted inhibition of the RAS pathway as a possible therapeutic approach to block MPNSTs

Numerous therapeutic options were explored to treat MPNSTs, with mostly negative or inconclusive results. Therefore, we developed a TC culture system derived from primary tumors that could be maintained over extended periods. This system preserves tumorigenic properties, including *Sox9* expression, rapid proliferation, and MPNST development after transplantation into nude mice. Using this system for drug screening, we used a library of 3500 annotated compounds, identifying 12 drugs that reduced *Sox9* expression. All were inhibitors directly or indirectly targeting the RAS pathway. This finding highly suggests a very strong link between the RAS pathway and *Sox9* expression and possibly GMT. The identified inhibitors target various nodes within the RAS pathway, including growth factor receptors and intracellular regulators. Therefore, rather than targeting a single branch of the RAS pathway, using a combination of compounds targeting multiple branches might constitute a way to completely silence the pathway, preventing *Sox9* expression and GMT, and possibly delaying or avoiding malignant transformation.

## MATERIALS AND METHODS

### Animals

Only male mice were considered in this study. Sex was not considered as a biological variable. Male mice used in this study were housed in a temperature- and humidity-controlled vivarium on a 12-hour dark-light cycle with free access to food and water. We bred *Prss56^Cre/Cre^*, *Rosa26^tdTom/tdTom^*, *Nf1^fl/fl^* mice with the *Nf1^fl/fl^* or *Nf1^+/−^* mice to obtain *Prss56^Cre/+^*, *Rosa26^tdTom/+^*, *Nf1^fl/fl^* or *Prss56^Cre/+^*, *Rosa26^tdTom/+^*, *Nf1^flox/−^* mutants, respectively. Genotyping strategies were described earlier (*7*). Information regarding mouse age and tumor stage is available in table S1. All animal manipulations were approved by a French Ethical Committee (APAFIS #19581) and were performed according to French and European Union regulations.

### Human samples

Tumor specimens were provided by NF1 patients undergoing surgical resections at Henri Mondor Hospital, Créteil, following written informed consent.

### Histopathology and immunohistochemistry

Tumors were dissected, immersion-fixed in 4% paraformaldehyde overnight at 4°C, and processed for paraffin embedding or cryo-embedding. Paraffin sections were cut at 5 μm and stained with hematoxylin and eosin (H&E) for histopathological evaluation. Cryosections were cut at 14 μm and immunolabeled with the following primary antibodies: rat anti-tdTomato (1:1000, Kerafast), rabbit anti–red fluorescent protein (RFP) (1:1000; Rockland), rabbit anti-S100B (1:500, Dako), rabbit anti-SOX10 (1:200, LSBio), rabbit anti-IBA1 (1:400, Wako), rabbit anti-CD3 (1:100, Abcam), rabbit anti-PH3 (1:200, Merck), rabbit anti-NGFR (1:500, Merck), rat anti-L1CAM (1:100, Merck), goat anti-CHL1 (1:100, R&D Systems), goat anti-PDGFRA (1:100, R&D Systems), goat anti-PDGFRB (1:100, R&D Systems), rabbit anti-SOX9 (1:500, Abcam), goat anti-CD34 (1:50, R&D Systems), rabbit anti-SPP1 (1:100, Abcepta), rabbit anti-CRABP1 (1:100, Merck), rabbit anti-H3K27me3 (1:1000, Cell Signaling), and rabbit anti-MIF (1:100, Abcam). Fluorophore-conjugated secondary antibodies were from Jackson ImmunoResearch. Briefly, sections were blocked/permeabilized for 1 hour at room temperature with immunoblock (IB) solution [4% bovine serum albumin (BSA)/0.3% Triton X-100 in phosphate-buffered saline (PBS)], then incubated overnight with the primary antibodies in IB at 4°C, washed, and incubated with the secondary antibodies for 2 hours at room temperature. Cell nuclei were counterstained with Hoechst (Life Technologies). Images were acquired on Zeiss LSM 900 confocal microscope and then processed and assembled using ImageJ and Photoshop.

### Sample processing and single-cell RNA sequencing

Mice were euthanized via isoflurane inhalation and perfused with PBS to remove circulating immune cells. Tumors were dissected and sampled for immunohistological and genomic analyses. Remaining tissue was minced into a slurry and digested in RPMI 1640 medium (Gibco, #42401-018) containing Liberase HD (0.25 mg/ml) (Roche, #05401089001) for 30 min at 37°C. Enzymatic digestion was halted by adding 10% fetal bovine serum (FBS) in RPMI (Gibco #10270106), and TC suspensions were collected by centrifugation (1200 rpm for 5 min). After mechanical dissociation and filtering (30 μm MACS SmartStratiners, Miltenyi Biotec, #130-098-458), single-cell suspensions were labeled with 4′,6-diamidino-2-phenylindole (DAPI) (Invitrogen, #D1306) and subjected to fluorescence-activated cell sorting (FACS) to recover viable cells.

NF1 patient tumor samples were excised from three ANNUBP and one MPNST, placed in ice-cold RPMI, and transported immediately to our laboratory for further processing. Information regarding patient datasets is provided in table S4. Human tumors were processed immediately after surgical resection using the same procedure as described above.

Live murine or human cells were isolated by FACS, and around 20,000 cells were loaded into one channel of the Chromium system using the V3 and V3.1 single-cell reagent kit (10x Genomics) to generate single-cell Gel Beads-in-emulsions (GEM). Following capture and lysis, cDNAs were synthesized and then amplified by PCR for 12 cycles as per the manufacturer’s protocol (10x Genomics). The amplified cDNAs were used to generate Illumina sequencing libraries that were each sequenced on one flow cell Nextseq500 Illumina.

### Bioinformatic analysis

BCL files were processed using 10x Genomics Cell Ranger software (version 2 or 3). For mouse PNSTs, reads were mapped onto a custom mouse transcriptome based on GRCm38 transcriptome, in which we added tdTomato sequence. For original human PNSTs, mapping was made on GRCh37 transcriptome. Raw count matrices were used for subsequent analyses. Publicly available scRNA-Seq datasets from human PNSTs were downloaded as filtered count matrices.

Count matrices were processed using RStudio and R Markdown to generate fully traceable notebooks, including the figures included in this article. To ensure version stability, a Singularity container containing R (version 3.6.3) and all packages required for the analyses was developed and used to compile notebooks (table S5). HTML reports, including the parameter values of all functions, and code to make the analyses and the figures, are available in a GitHub repository and duplicated on Zenodo (see the “Data and materials availability” section).

From the count matrices, datasets were analyzed individually using Seurat V3 package (*32*). Cells having less than exp(6) (403) Unique Molecular Identifiers (UMI) or less than 600 genes were filtered out. Doublets were removed using the scDblFinder tool (*33*) and scds in hybrid mode (*34*). Then, cells having more than 50% of UMI related to mitochondrial genes or more than 30% related to ribosomal genes were filtered out. The UMI count matrix for remaining cells was log-normalized using the Seurat::NormalizeData function. Cell cycle annotation was made using Seurat::AddCellCycle. Single cells were annotated for cell type using the Seurat::AddModuleScore function, and cell type–specific marker gene sets were listed in table S6 when annotating all cells or in table S7 for the Fb dataset.

The same annotation procedure was applied to the human datasets, with an additional analysis of CNVs using the infercnv package to assess the annotation of TCs, using macrophages as reference.

Individual datasets were merged to generate combined datasets. For the human PNST atlas, the intersection of Ensembl gene identifiers between original and published datasets was used to homogenize count matrices. For each individual dataset, cells were extracted based on cell type annotation and then merged to generate the TC- and Fb-specific datasets. Seurat::PCA and harmony (*35*) or FastMNN (*36*) was used to remove batch effect. Cell projections were made using Seurat::RunUMAP, Seurat::RunTSNE, or DM from the destiny package (*37*). Batch-effect correction and projection methods are summarized in Table 1.

**Table 1.** Batch-effect correction method and projection for each dataset. The first column corresponds to the name of the dataset, as mentioned in the main text. The batch-effect correction method and projection depends on the cell types present in the dataset.

Dataset	Cell type	Batch-effect correction	Projection
Pan-PNST atlas	All	PCA—harmony	tSNE
Pan-PNST atlas	Tumor cells	FastMNN	DM—UMAP
Pan-PNST atlas	Fibroblasts	FastMNN	UMAP
MPNST atlas	All	PCA—harmony	tSNE
Primary/transplanted	Tumor cells	FastMNN	UMAP
Human PNST atlas	All	PCA—harmony	tSNE
Human PNST atlas	Tumor cells	FastMNN	UMAP

Trajectory inference was made using two distinct methods: slingshot (*14*) and TinGa (*38*), using cells from the pNF datasets as trajectory root. For the functional analysis, the TCs were divided into three subsets using *k*-means function from the stats base package, on the batch-effect corrected space. Differential expression was conducted using Seurat::FindMarkers. Heatmaps were made using the ComplexHeatmap package (*39*). Functional enrichment analyses were conducted using the msigdbr package (https://CRAN.R-project.org/package=msigdbr) for the gene set database, and clusterProfiler package for the analyses (*40*).

### Whole-exome sequencing

Raw sequences were demultiplexed to FASTQ, taking care of UMIs, with bcl2fastq v2.20.0.422 using default parameters. Raw reads were trimmed for bad quality bases, adapter content, and polymere tracks using fastp v0.23.2. General reads QC were assessed using fastqc v0.12.1, sample contamination with FastQ_Screen v0.15.3, and results were aggregated with multiQC v1.13. Mapping against the murine genome UCSC mm10 was performed with bwa-mem2 v.2.2.1 with default parameters, except for “-A 2 -E 1.” Mapped reads QC was performed using samtools stats/flagstat/idxstats v1.18, and coverage was assessed using mosdepth v0.2.6. Deduplication of reads because of UMIs was performed using umi_tools v1.1.2. PCR and optical duplicate marking were performed using Picard tools v2.23.5. Somatic variant calling was performed using the GATK mutect2 v4.2.6.1 with recommended guidelines (*41*). Variant annotation and filtering were performed using snpEff/snpSift v4.3.1t (*42*). Copy number analysis of tumor/normal pairs was performed using EaCoN v0.2.8 using the ASCAT2 segmentation method, with default parameters (*43*).

### TC culture

Cells dissociated from mouse MPNST were frozen in 90% FBS/10% DMSO and stored at −150°C. Once needed, vials were thawed by gentle agitation in a water bath at 37°C, DMSO was removed, and cells were resuspended and cultured in Dulbecco’s modified Eagle’s medium (DMEM), 10% FBS, and 1% penicillin/streptomycin in T75 flask (Thermo Fisher Scientific). As soon as the primary cells reached confluence, they were subcultured in a new T75 flask by 1:10 dilutions, twice a week.

### shRNA-mediated *Sox9* KD

Lentivirus encoding shRNA targeting *Sox9* (TRCN0000321420), *Cxcl12* (TRCN0000184347), *Pdgfra* (TRCN0000321928), *Pdgfrb* (TRCN0000321931), *Crabp1* (TRCN0000011959), and *Mif* (TRCN0000067344) was obtained from Sigma-Aldrich. Viral infection was carried out as described by the manufacturer (MISSION Lentiviral Transduction Particles) at MOI (multiplicity of infection) 5. Briefly, murine primary MPNST cells were seeded at 5 × 10^3^ in 96-well plates and transduced 24 hours later when wells became 50% confluent. Incubation with the virus was carried out overnight in 110 μl of DMEM containing 10% FBS, 1% penicillin/streptomycin, and hexadimethrine bromide (8 μg/ml). Transduced cells were selected in medium containing puromycin (3 μg/ml) for 72 hours and then cultured in the presence of puromycin (1.5 μg/ml) during clones’ expansion. Nontargeting lentivirus (SHC201V and SHC201V, Sigma-Aldrich) was used as control.

### Tumor grafting

Approximately 10,000 of Tom^+^ TCs from each primary tumor were subcutaneously grafted into nude female recipients under isoflurane anesthesia. After monitoring for 2 weeks, tumors were extracted and dissociated, and a fraction (10,000 cells) were regrafted using the same procedure.

### siRNA-mediated Sox9 KD

For siRNA KD targeting *Sox9* (ON-TARGETplus Mouse Sox9 siRNA, L-059108-01-0005, Horizon Discovery) in MPNST cultured cells, we used Lipofectamine RNAiMAX (Life Technologies) following the manufacturer’s instructions. Nontargeting siRNA (ON-TARGETplus Non-targeting Pool, D-001810-10-05) was used as control.

### RNA preparation and qRT-PCR

Total RNAs were isolated using the RNAqueous-micro kit (Ambion). After deoxyribonuclease treatment, concentration of RNA was determined using NanoDrop and QuBit technology. cDNA was synthesized using the Maxima First Strand cDNA Synthesis Kit, and qRT-PCR was performed using the Maxima SYBER green/ROX qPCR Master Mix X2 (both from Thermo Fisher Scientific) and amplified using the StepOnePlus Real-Time PCR (Applied Biosystems). The relative abundance values of each amplification product were normalized to the internal controls (cyclophilin, tubulin, and actin). Dedicated PCR primers are reported in Table 2.

**Table 2.** PCR primer sequences. F, forward; R, reverse.

*Actin*	F	TGTTACCAACTGGGACGACA
	R	GGGGTGTTGAAGGTCTCAAA
*Tubulin*	F	CGGGTCTCCAGGGCTTCT
	R	GGGCTGGGTAAATGGAGA
*Cyclophilin*	F	CCATCGTGTCATCAAGGACTT
	R	TTGCCATCCAGCCAGGAGGTC
*Sox9*	F	AGGAAGTCGGTGAAGAACGG
	R	GGACCCTGAGATTGCCCAGA
*Cxcl12*	F	GCCAACGTCAAGCATCTGAA
	R	TGGGCTGTTGTGCTTACTTG
*Pdgfra*	F	ACTTCGTGGACAGTTTTGGC
	R	ATCACCAACAGCACCAACAC
*Pdgfrb*	F	ATCGCGCCACTTAATAAC
	R	TCCCTCATGATGTCTCGAGC
*Mif*	F	GTGAACACCAATGTTCCCCG
	R	TGCACTGCGATGTACTGTGC
*Crabp1*	F	GGACGCAAATGCAGGAGTTT
	R	AGCTCTCGGGTCCAGTAAGT

### High-content drug screening

#### Compound annotated set library

The Annotated Set library is composed of the following individual compound libraries: CLOUD (CeMM Library of Unique Drugs, Enamine), Prestwick Chemical Library (Prestwick Chemical), Tocriscreen Plus (Tocris), and SGC Chemical Probes. Compounds were stored in Echo 384-LDV plates (LP-0200) as 10 mM stock solutions in DMSO. All compounds are listed in table S8.

#### Compound screening

MPNST cells were seeded at a concentration of 800 cells per well onto 384-well PhenoPlate microplates (6057302, Revvity) and incubated for 24 hours at 37°C, 5% CO_2_ before compound treatment. Compounds were dispensed onto cells using an Echo acoustic dispenser (Beckman Coulter Life Sciences) at a final concentration of 10 μM for the primary screen and incubated for 12 hours. Dose-response experiments were performed at 8 concentrations: 30 nM, 100 nM, 300 nM, 1 μM, 3 μM, 6 μM, 10 μM (repeat of the primary screen concentration), and 30 μM. Treatment was followed by fixation by addition of equal volume of 8% paraformaldehyde (Sigma-Aldrich) and 20-min incubation, blocking, and permeabilization (0.3% Triton X-100, 4% BSA in PBS), incubation with anti-Sox9 primary antibody (1:1000 in blocking buffer, ab185230, Abcam), and further incubation with secondary antibody [1:1000, Goat anti-Rabbit IgG (H+L) Cross-Adsorbed Secondary Antibody, Alexa Fluor 488, A11008, Thermo Fisher Scientific] and DAPI (1:1000, Thermo Fisher Scientific) in blocking buffer. All incubations of the staining protocol were carried out at room temperature for an hour unless otherwise specified. Wells were washed using PBS in between staining solutions. Plates were imaged using an IN Cell Analyzer 2200 (GE Healthcare Life Sciences). Six fields of view were acquired per well, using the 20× objective (0.45 numerical aperture). Images were imported into an OMERO Plus database and analyzed using CellProfiler. Data were normalized to DMSO control wells (16 per plate) within the same plate. Two independent experiments were performed for both the primary screen (single technical replicate) and dose-response confirmation test (two technical replicates). Compounds were considered potential hits if reducing SOX9 intensity under 80% of the DMSO control in one of the two replicates of the primary screen with more than 50 cells analyzed, or in both replicates with an average ≥100 cells analyzed per independent experiment. Hit reconfirmation was determined by similar reduction of SOX9 intensity at screening concentration (10 μM) in the dose-response experiment.
