# Supplementary material for: Glial-to-mesenchymal transition of tumor Schwann cells drives the genetic burden in MPNSTs from neurofibromatosis type 1 mouse model
Source: Sci Adv. 2025 Nov 12;11(46):eadt9210. doi: 10.1126/sciadv.adt9210 (PMC12609162; doi:10.1126/sciadv.adt9210)
Supplement: Supplementary file 1 — Figs. S1 to S6 Legend for tables S1 to S8 [file sciadv.adt9210_sm.pdf]

Science Advances
AAAS

Supplementary Materials for

Glial-to-mesenchymal transition of tumor Schwann cells drives the genetic burden in MPNSTs from neurofibromatosis type 1 mouse model

Katarzyna J. Radomska *et al.*

Corresponding author: Piotr Topilko, piotr.topilko@inserm.fr

Sci. Adv. **11**, eadt9210 (2025)
DOI: 10.1126/sciadv.adt9210

The PDF file includes:

Figs. S1 to S6
Legend for tables S1 to S8

Other Supplementary Material for this manuscript includes the following:

Tables S1 to S8

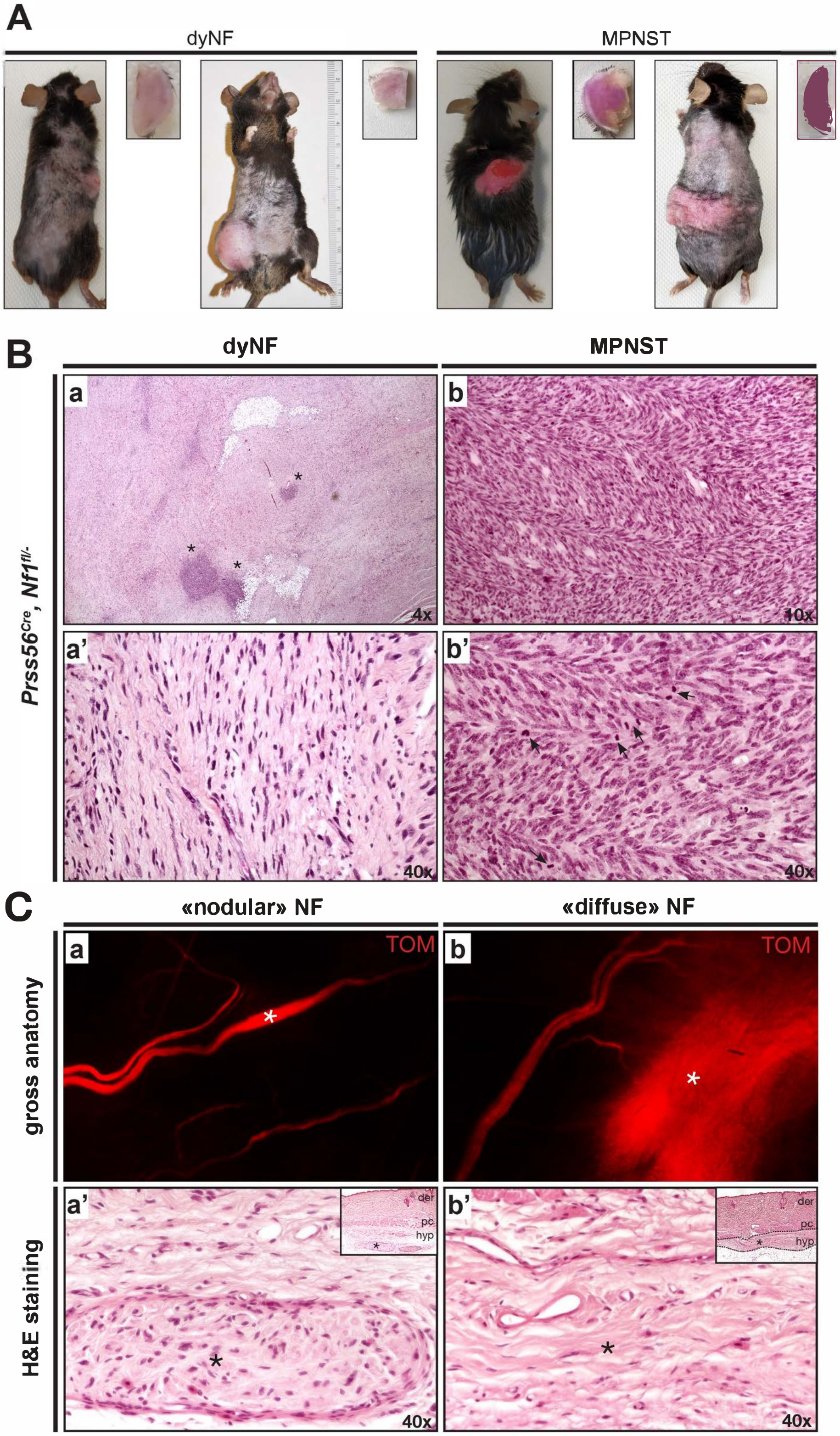
A
dyNF
MPNST
B
dyNF
MPNST
Prss56Cre, Nf1fl/-
a
4x
b
10x
a'
40x
b'
40x
C
«nodular» NF
«diffuse» NF
gross anatomy
a
TOM
b
TOM
H&E staining
a'
der
pc
hyp
40x
b'
der
pc
hyp
40x

Figure S1. Spectrum of peripheral nerve sheath tumors in Nf1 mutant mouse model, related to Figure 1. (A) Gross anatomy of dyNF and MPNST-bearing *Prss56*^{Cre}, *Nf1*$^{fl/-}$ males. Dissected tumors are shown on the right. **(B)** H&E staining of dyNF (a, a') and MPNST (b, b') from *Prss56*^{Cre}, *Nf1*$^{fl/-}$ mice at two magnifications. Asterisks in (a) indicate clusters of mononuclear cells. Arrows in (b') indicate mitotic cells. **(C)** Comparison of "nodular" (a, a') and "diffuse" (b, b') pNFs (asterisks) in *Prss56*^{Cre}, *Nf1*$^{fl/fl}$ mice. Endogenous Tomato (TOM) fluorescence is shown in (a) and (b), and H\&E staining of the corresponding lesions in (a') and (b'). Insets represent low magnification images showing tissue architecture and localization of respective pNFs. der: dermis, hypo: hypodermis, pc: panniculus carnosus muscle.

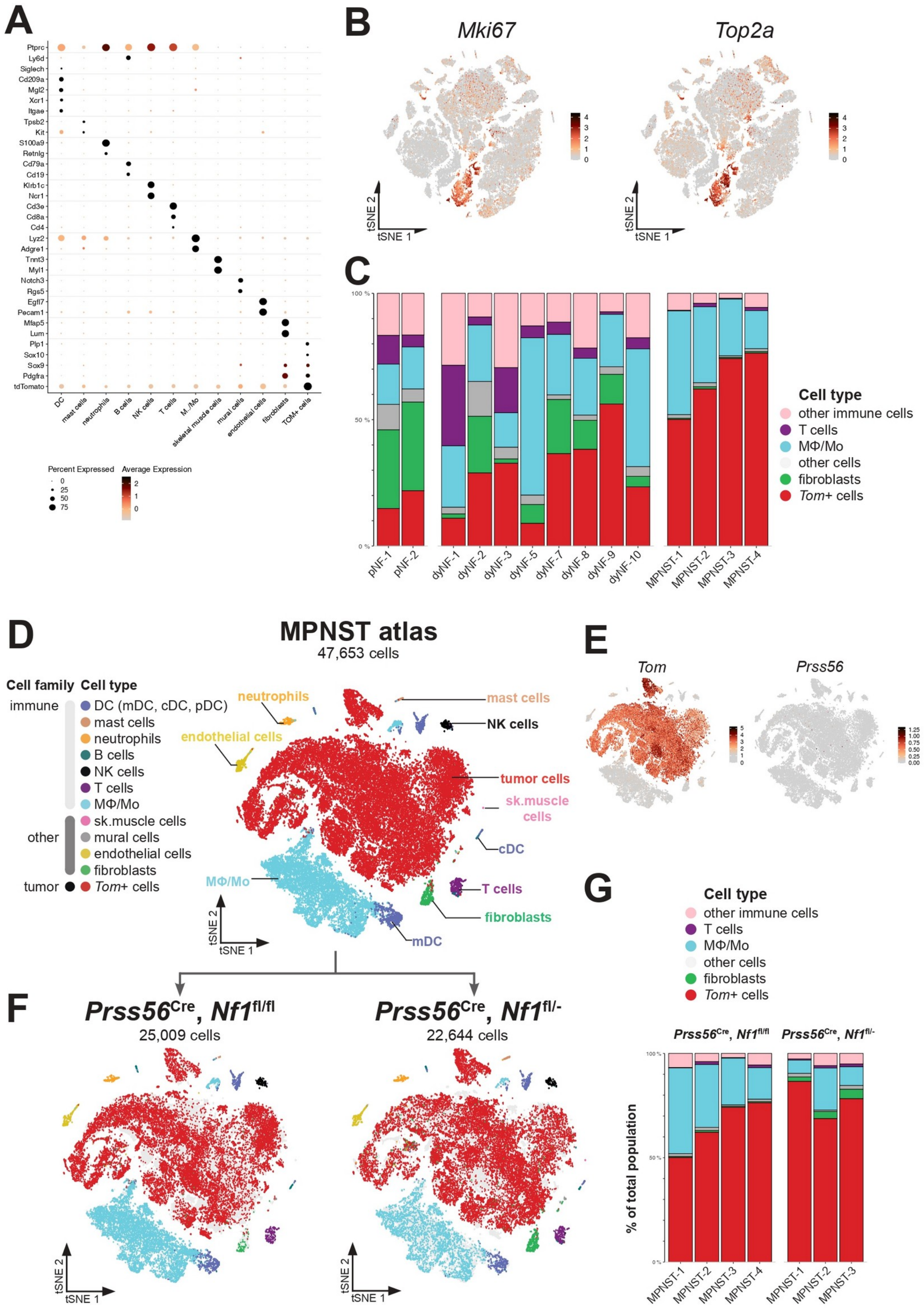
A
Ptprc
Ly6d
Siglech
Cd209a
Mgl2
Xcr1
Itgae
Tpsb2
Kit
S100a9
Retnlg
Cd79a
Cd19
Klrb1c
Ncr1
Cd3e
Cd8a
Cd4
Lyz2
Adgre1
Tnnt3
Myl1
Notch3
Rgs5
Egfl7
Pecam1
Mfap5
Lum
Plp1
Sox10
Sox9
Pdgfra
tdTomato
DC
mast cells
neutrophils
B cells
NK cells
T cells
M.../Mo
skeletal muscle cells
mural cells
endothelial cells
fibroblasts
TOM+ cells
Percent Expressed
Average Expression
0
25
50
75
B
Mki67
Top2a
tSNE 2
tSNE 1
C
100 %
50 %
0 %
pNF-1
pNF-2
dyNF-1
dyNF-2
dyNF-3
dyNF-5
dyNF-7
dyNF-8
dyNF-9
dyNF-10
MPNST-1
MPNST-2
MPNST-3
MPNST-4
Cell type
other immune cells
T cells
MΦ/Mo
other cells
fibroblasts
Tom+ cells
D
MPNST atlas
47,653 cells
Cell family
Cell type
immune
DC (mDC, cDC, pDC)
mast cells
neutrophils
B cells
NK cells
T cells
MΦ/Mo
other
sk.muscle cells
mural cells
endothelial cells
fibroblasts
tumor
Tom+ cells
neutrophils
mast cells
NK cells
endothelial cells
tumor cells
sk.muscle cells
cDC
MΦ/Mo
T cells
fibroblasts
mDC
tSNE 2
tSNE 1
E
Tom
Prss56
F
Prss56Cre, Nf1fl/fl
25,009 cells
Prss56Cre, Nf1fl/-
22,644 cells
tSNE 2
tSNE 1
G
Cell type
other immune cells
T cells
MΦ/Mo
other cells
fibroblasts
Tom+ cells
Prss56Cre, Nf1fl/fl
Prss56Cre, Nf1fl/-
% of total population
100 %
50 %
0 %
MPNST-1
MPNST-2
MPNST-3
MPNST-4
MPNST-1
MPNST-2
MPNST-3

Figure S2. Characterization of cellular composition of PNSTs from *Nf1* mutant mice, related to Figure 2. (A) Dotplot of average expression of markers of the different cell types composing the PNSTs. Dot size represents the percentage of cells in the tumor and color scale represents the average expression level across all cells within population. **(B)** tSNE plot of Pan MPNST atlas overlaid with the expression of cell cycle-related genes *Mki67* and *Top2a*. **(C)** Barplot depicting the cellular composition of individual datasets included in the Pan PNST atlas. Note that datasets dyNF_4 and dyNF_6 are not shown because they originate from FACS sorting of TOM+ cells. **(D)** MPNST transcriptomic atlas assembled from *Prss56*^{Cre}, *Nf1*$^{fl/fl}$ and *Prss56*^{Cre}, *Nf1*$^{fl/-}$ primary MPNST datasets. Results are presented as tSNE plot to visualize cellular heterogeneity. Expression score-based annotation resolved 12 distinct cell populations. **(E)** tSNE plot of MPNST atlas overlaid with expression of *Tom* and *Prss56*. Cells are color-coded according to gene expression level. **(F)** tSNE plot of MPNST atlas split by genotype. Cells from the complete atlas are shown as a light grey background. **(G)** Barplot depicting the cellular composition of individual MPNSTs included in the MPNST atlas.

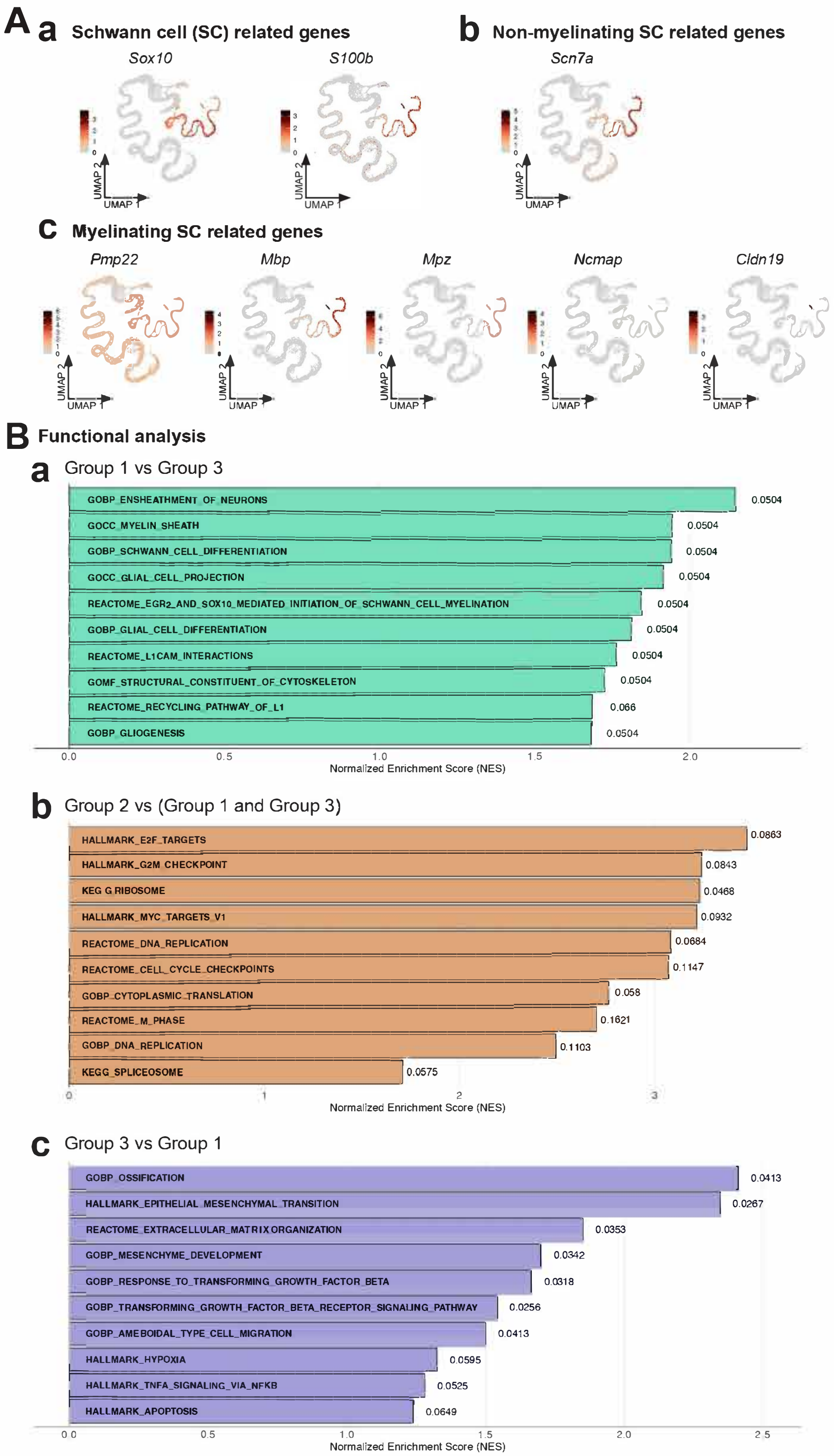

A
a Schwann cell (SC) related genes
Sox10
S100b
b Non-myelinating SC related genes
Scn7a
c Myelinating SC related genes
Pmp22
Mbp
Mpz
Ncmap
Cldn19
UMAP 1
UMAP 2
B Functional analysis
a Group 1 vs Group 3
GOBP_ENSHEATHMENT_OF_NEURONS 0.0504
GOCC_MYELIN_SHEATH 0.0504
GOBP_SCHWANN_CELL_DIFFERENTIATION 0.0504
GOCC_GLIAL_CELL_PROJECTION 0.0504
REACTOME_EGR2_AND_SOX10_MEDIATED_INITIATION_OF_SCHWANN_CELL_MYELINATION 0.0504
GOBP_GLIAL_CELL_DIFFERENTIATION 0.0504
REACTOME_L1CAM_INTERACTIONS 0.0504
GOMF_STRUCTURAL_CONSTITUENT_OF_CYTOSKELETON 0.0504
REACTOME_RECYCLING_PATHWAY_OF_L1 0.066
GOBP_GLIOGENESIS 0.0504
0.0 0.5 1.0 1.5 2.0
Normalized Enrichment Score (NES)
b Group 2 vs (Group 1 and Group 3)
HALLMARK_E2F_TARGETS 0.0863
HALLMARK_G2M_CHECKPOINT 0.0843
KEGG_RIBOSOME 0.0468
HALLMARK_MYC_TARGETS_V1 0.0932
REACTOME_DNA_REPLICATION 0.0684
REACTOME_CELL_CYCLE_CHECKPOINTS 0.1147
GOBP_CYTOPLASMIC_TRANSLATION 0.058
REACTOME_M_PHASE 0.1621
GOBP_DNA_REPLICATION 0.1103
KEGG_SPLICEOSOME 0.0575
0 1 2 3
Normalized Enrichment Score (NES)
c Group 3 vs Group 1
GOBP_OSSIFICATION 0.0413
HALLMARK_EPITHELIAL_MESENCHYMAL_TRANSITION 0.0267
REACTOME_EXTRACELLULAR_MATRIX_ORGANIZATION 0.0353
GOBP_MESENCHYME_DEVELOPMENT 0.0342
GOBP_RESPONSE_TO_TRANSFORMING_GROWTH_FACTOR_BETA 0.0318
GOBP_TRANSFORMING_GROWTH_FACTOR_BETA_RECEPTOR_SIGNALING_PATHWAY 0.0256
GOBP_AMEBOIDAL_TYPE_CELL_MIGRATION 0.0413
HALLMARK_HYPOXIA 0.0595
HALLMARK_TNFA_SIGNALING_VIA_NFKB 0.0525
HALLMARK_APOPTOSIS 0.0649
0.0 0.5 1.0 1.5 2.0 2.5
Normalized Enrichment Score (NES)

Figure S3. Functional characterization of tumor cells extracted from the murine Pan-PNST atlas, related to Figure 3. (A) UMAP plot of tumor cells overlaid with the expression Schwann cell (SC) (a), non-myelinating SC (b) and myelinating SC (c) related transcripts. **(B)** Functional enrichment analyses of each group shown in Figure 3Ae. Group 1 was compared to group 3 (a) and conversely (c). Group 2 was compared to both groups 1 and 3 and depicts enrichment in cell-cycle related genes (b).

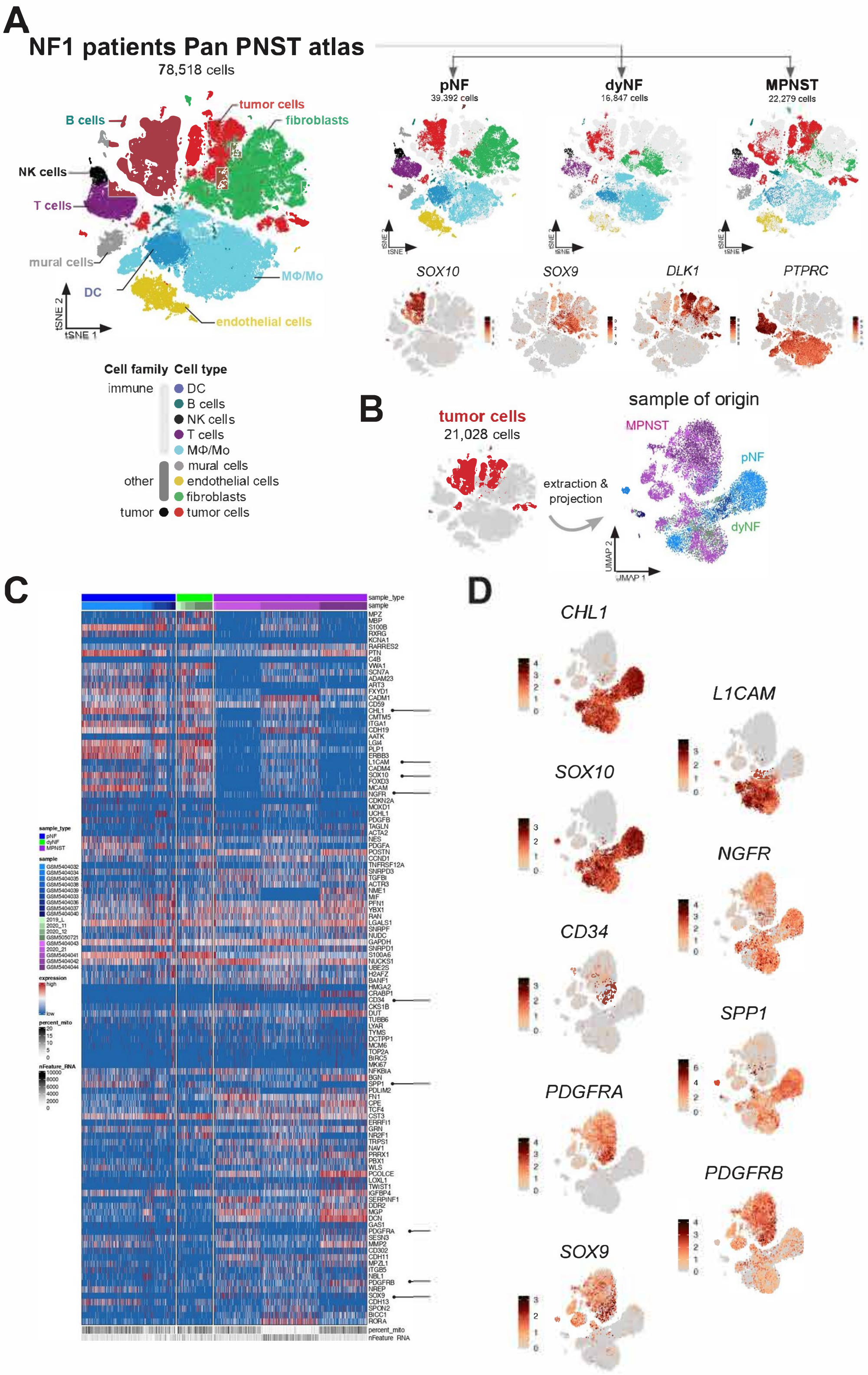
A
NF1 patients Pan PNST atlas
78,518 cells
tumor cells
B cells
fibroblasts
NK cells
T cells
mural cells
DC
MΦ/Mo
endothelial cells
tSNE 2
tSNE 1
pNF
39,392 cells
dyNF
16,847 cells
MPNST
22,279 cells
SOX10
SOX9
DLK1
PTPRC
Cell family
Cell type
immune
other
tumor
DC
B cells
NK cells
T cells
MΦ/Mo
mural cells
endothelial cells
fibroblasts
tumor cells
B
tumor cells
21,028 cells
sample of origin
MPNST
pNF
dyNF
extraction &
projection
UMAP 2
UMAP 1
C
sample_type
sample
percent_mito
nFeature_RNA
D
CHL1
L1CAM
SOX10
NGFR
CD34
SPP1
PDGFRA
PDGFRB
SOX9

Figure S4. Single-cell phenotyping of human PNSTs, related to Figure 3. (A) Pan PNST atlas assembled from 4 original and 14 published NF1 patient datasets (accession number GSE165826 and GSE179043, Table S2). (Left panel) Data are presented as tSNE plot to visualize cellular heterogeneity. Expression score-based annotation resolved nine distinct cell populations. (Right panel) tSNE plot of NF1 patient PNSTs split by tumor type. Cells from the complete atlas are shown as a light grey background. On bottom, cells are color-coded according to gene expression level. **(B)** Tumor cells are extracted from NF1 patient PNSTs atlas, projected using UMAP and colored based on tumor type. **(C)** Heatmap showing transcriptomic changes in tumor cells during pNF-to-MPNST progression, for the same genes identified in *Prss56*^{Cre}, *Nf1*$^{fl/fl}$ primary tumors. Cells are ordered by tumor types and samples of origin (top annotation). Bottom annotation shows quality metrics: proportion of expression levels related to mitochondrial genes (percent.mito) and total number of genes detected by cell (nFeature_RNA). Note that fewer genes are detected in human scRNA-Seq compared to mice. **(D)** UMAP plot of NF1 patient tumor cells overlaid with the expression of genes validated at proteomic level in *Prss56*^{Cre}, *Nf1*$^{fl/fl}$ primary tumors.

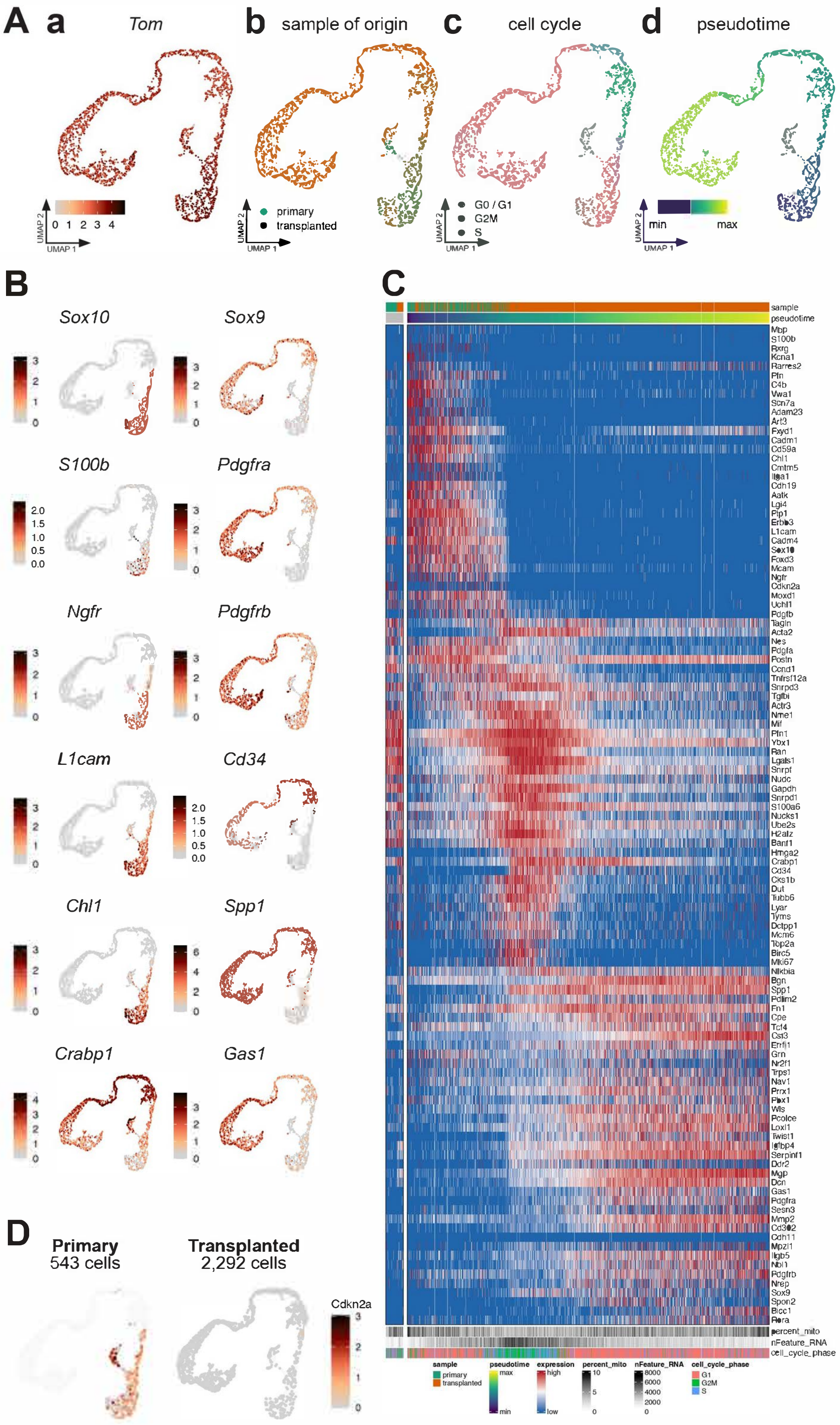

A a Tom
b sample of origin
c cell cycle
d pseudotime
primary
transplanted
G0 / G1
G2M
S
min
max
UMAP 1
UMAP 2
B
Sox10
Sox9
S100b
Pdgfra
Ngfr
Pdgfrb
L1cam
Cd34
Chl1
Spp1
Crabp1
Gas1
C
sample
pseudotime
percent_mito
nFeature_RNA
cell_cycle_phase
expression
high
low
G1
G2M
S
D
Primary
543 cells
Transplanted
2,292 cells
Cdkn2a

Figure S5. Dysplastic NF allografts recapitulate GMT program, related to Figure 3. **(A)** Tumor cells from Nude mice transplanted with a primary dyNF (*Prss56*^{Cre}, *Nf1*$^{fl/fl}$ mouse) were projected using UMAP. Cells were colored based on Tom expression (a), sample of origin (b), cell cycle phase annotation (c) and pseudotime inferred with slingshot (d). Gray cells in (d) did not belong to the linear trajectory branch inferred with slingshot and have no pseudotime value. **(B)** UMAP plot of tumor cells overlaid with the expression of genes validated at protein level in *Prss56*^{Cre}, *Nf1*$^{fl/fl}$ primary tumors. **(C)** Heatmap showing transcriptomic changes between primary and transplanted tumors. Cells were ordered by increasing pseudotime values. The top annotation shows pseudotime and sample of origin. Cells without pseudotime value were arbitrary placed on the right, as separated group. Bottom annotation shows cell cycle annotation and quality metrics: proportion of expression levels related to mitochondrial genes (percent.mito) and total number of genes detected by cell (nFeature_RNA). Note that cells without pseudotime values, in the left column, are characterized by high expression of mitochondrial genes, associated with low number of genes detected. **(D)** UMAP plot split by sample overlaid with the expression of *Cdkn2a*. Cells from the complete dataset are shown as a light grey background.

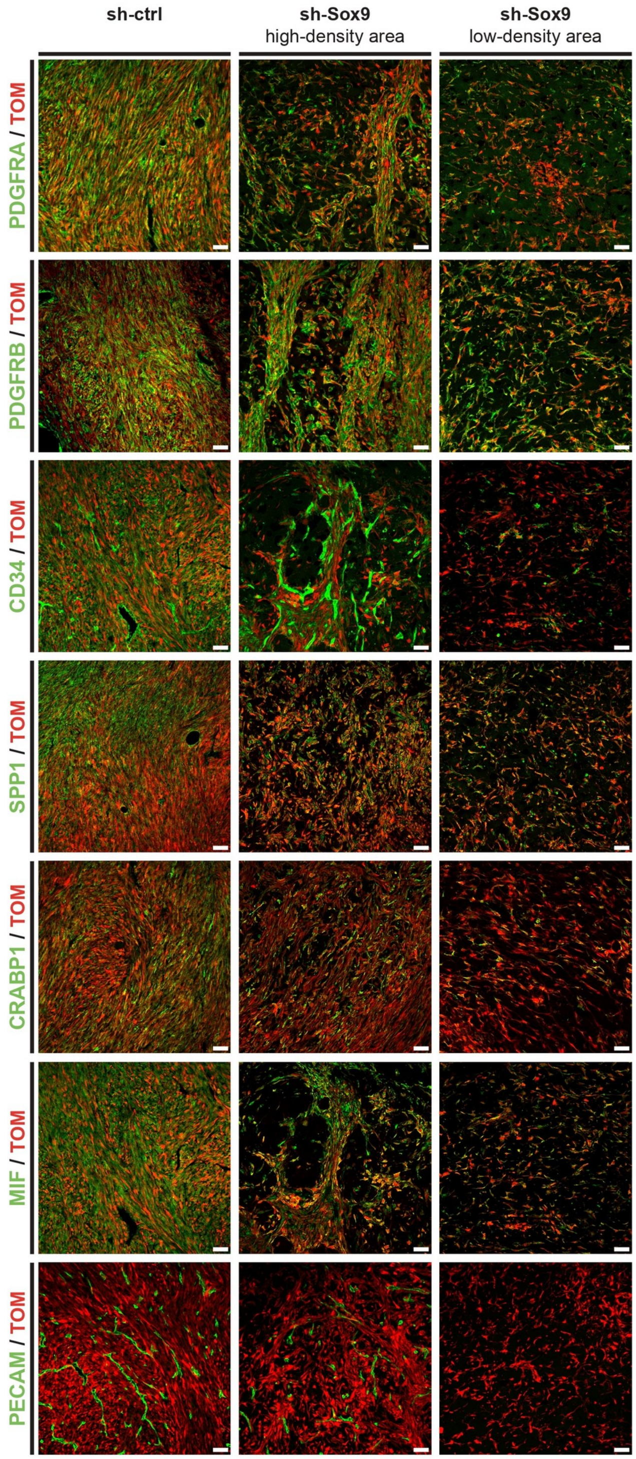

Figure S6. *Sox9* knockdown impact the expression of mesenchymal markers in vivo, related to Figure 6. IHC of tumors developed in nude mice transplanted as indicated above (sh-ctrl, sh-Sox9, two areas) and labeled for TOM (tumor cells) along with PDGFRA, PDGFRB, CD34, SPP1, CRABP1, MIF and PECAM. Scale bar = 50µm.

Other Supplementary Materials for this manuscript include the following:

Table S1. Mouse features, tumor histological characterization, and NGS identifiers annotation for all Nf1 mutant mice
Table S2. Information regarding NF1 patient scRNA-Seq datasets
Table S3. Efficacy of stable inhibition induced by shRNA treatment and measured by qRT-PCR (n=4 for each treatment)
Table S4. Annotation of top 12 selected compounds after drug screening
Table S5. Version of R packages used for scRNA-Seq data analysis
Table S6. Gene markers used for cell type annotation in all scRNA-Seq datasets
Table S7. Gene markers used for cell type annotation in the fibroblast dataset
Table S8. Information about the 3500 compounds involved in the screening

These tables are provided as Excel or CSV files.
